# New Chemical Probe Targeting Bacterial NAD Kinase

**DOI:** 10.3390/molecules25214893

**Published:** 2020-10-22

**Authors:** David A. Clément, Clarisse Leseigneur, Muriel Gelin, Dylan Coelho, Valérie Huteau, Corinne Lionne, Gilles Labesse, Olivier Dussurget, Sylvie Pochet

**Affiliations:** 1 Institut Pasteur, Unité de Chimie et Biocatalyse, UMR3523 CNRS, 75015 Paris, France; david.clement@pasteur.fr (D.A.C.); dylan.coelho@etu.parisdescartes.fr (D.C.); valerie.huteau@pasteur.fr (V.H.); 2 Faculté des Sciences, Université de Paris, Sorbonne Paris Cité, 75013 Paris, France; clarisse.leseigneur@pasteur.fr; 3 Institut Pasteur, Unité de Recherche Yersinia, 75015 Paris, France; 4 Centre de Biochimie Structurale (CBS), CNRS, INSERM, Université de Montpellier, 34090 Montpellier, France; gelin@cbs.cnrs.fr (M.G.); lionne@cbs.cnrs.fr (C.L.)

**Keywords:** antibiotics, chemical probe, diadenosine derivatives, NAD kinase, nucleoside, Sonogashira coupling, staphylococci

## Abstract

Nicotinamide adenine dinucleotide (NAD) kinases are essential and ubiquitous enzymes involved in the tight regulation of NAD/nicotinamide adenine dinucleotide phosphate (NADP) levels in many metabolic pathways. Consequently, they represent promising therapeutic targets in cancer and antibacterial treatments. We previously reported diadenosine derivatives as NAD kinase inhibitors with bactericidal activities on *Staphylococcus aureus*. Among them, one compound (namely **NKI1**) was found effective in vivo in a mouse infection model. With the aim to gain detailed knowledge about the selectivity and mechanism of action of this lead compound, we planned to develop a chemical probe that could be used in affinity-based chemoproteomic approaches. Here, we describe the first functionalized chemical probe targeting a bacterial NAD kinase. Aminoalkyl functional groups were introduced on **NKI1** for further covalent coupling to an activated Sepharose^TM^ matrix. Inhibitory properties of functionalized **NKI1** derivatives together with X-ray characterization of their complexes with the NAD kinase led to identify candidate compounds that are amenable to covalent coupling to a matrix.

## 1. Introduction

Nicotinamide adenine dinucleotide (NAD) kinases (NADK) are ubiquitous enzymes, which catalyze the phosphorylation of NAD to nicotinamide adenine dinucleotide phosphate (NADP), which is subsequently reduced to NADPH [1,2,3]. Since it is the only known enzyme producing NADP de novo, NAD kinase plays a crucial role in controlling the intracellular balance of NAD(H) and NADP(H) in many cellular metabolic pathways [4,5]. While the NADK enzymatic activity has been known for decades, their genes were identified more recently, leading to the discovery of orthologs in nearly all living organisms. The essentiality of the NADK gene has been experimentally validated in several bacteria, including *Escherichia coli* [6], *Salmonella enterica, Bacillus subtilis* [7,8], *Mycobacterium tuberculosis* [9] and *Staphylococcus aureus* [10,11,12]. Moreover, it was shown that human NAD kinase displays kinetic and structural features that differ considerably from that of prokaryotes [13,14]. Therefore, NADK represents a promising target for the development of novel antibiotics with an original mode of action.

**Figure 1:**
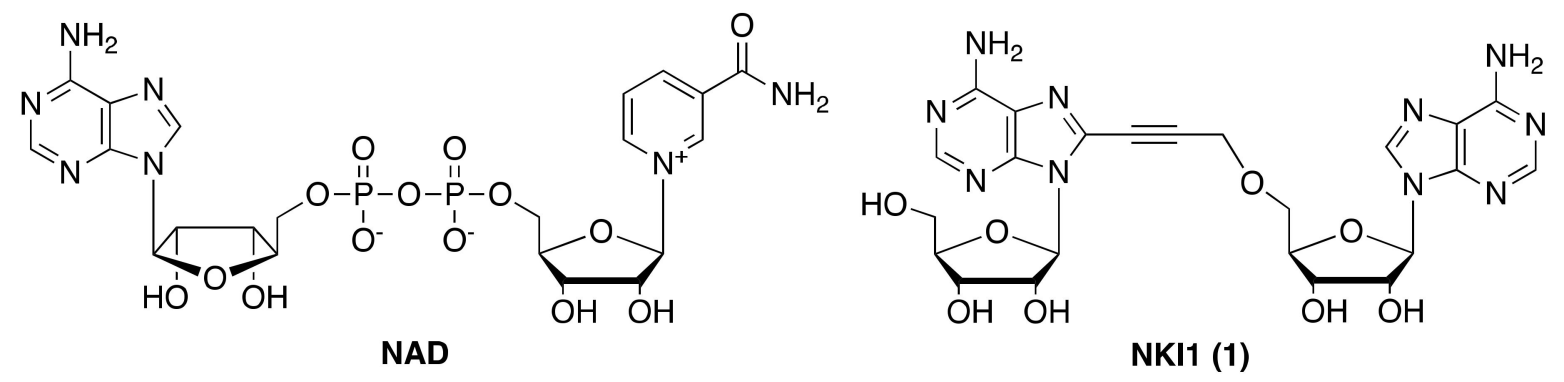
Chemical structures of nicotinamide adenine dinucleotide (NAD) and NAD kinase inhibitor NKI1 (**1**).

Previously we solved the crystal structures of NADK from *Listeria monocytogenes* (*Lm*NADK1) in the free state and bound to NAD and NADP, and described the first non-natural inhibitor of this enzyme [15]. Following a fragment-based approach, we identified a series of diadenosine derivatives with low micromolar inhibitory potencies against recombinant *Lm*NADK1 and *S. aureus* NADK [16,17]. We subsequently discovered the first NAD kinase inhibitor (namely **NKI1**, Figure 1) active in mice infected with *S. aureus*, including antibiotic-resistant strains [18].

To get more information about the selectivity of our lead compound and its mechanism of action, we aimed at immobilizing **NKI1** derivatives on a Sepharose^TM^ matrix that could be further used in chemical proteomics [19]. Affinity chromatography is one of the most powerful chromatographic methods to analyze and purify proteins from complex mixtures or crude extracts. Various immobilized-nucleos(t)ides and cofactors are now commercially available. However specific affinity matrices may need to be developed by coupling the suitable ligand onto the matrix. The attachment chemistry and the spacer between the ligand and the matrix are important factors to be considered [20].

**Figure 2:**
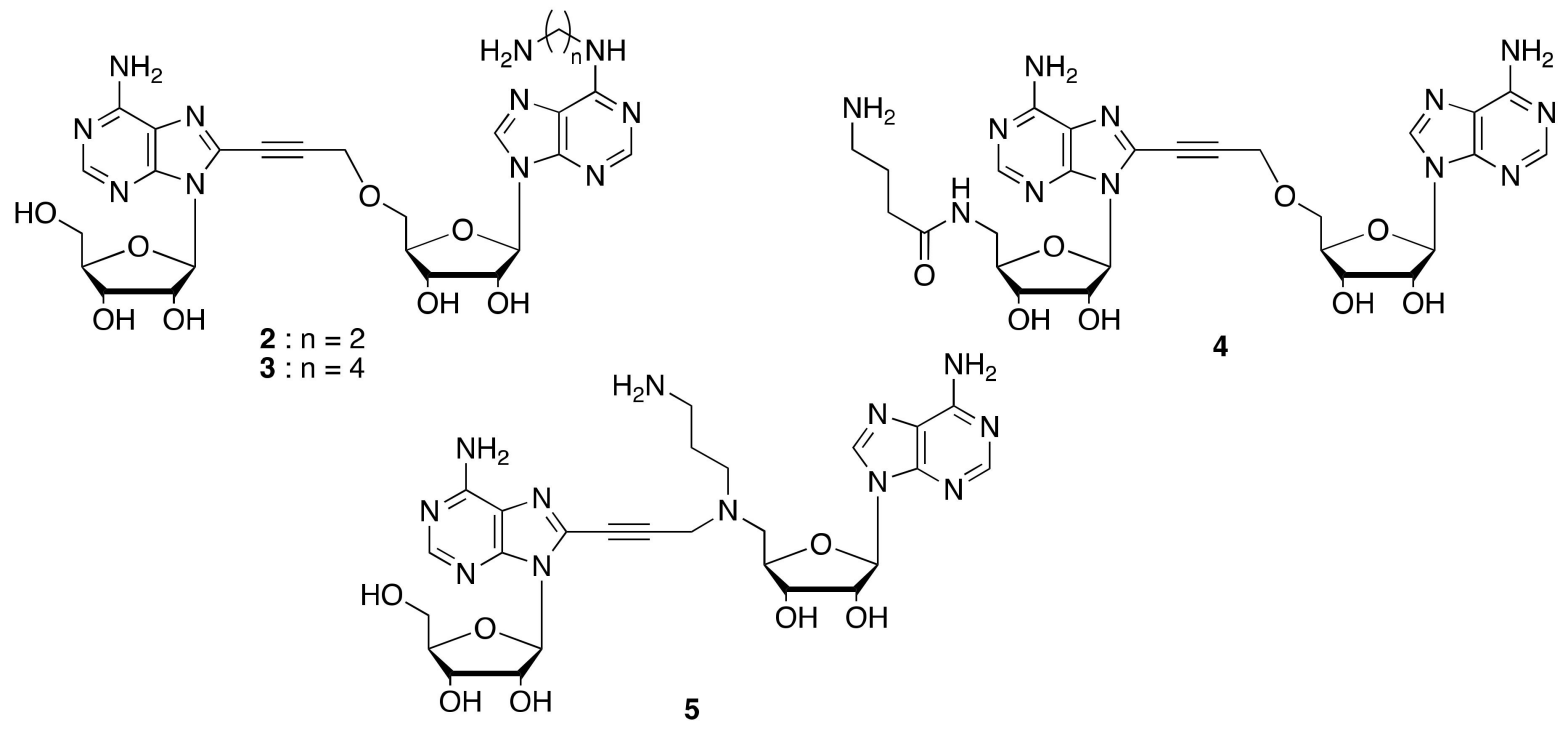
Chemical structures of the **NKI1** analogues **2**–**5**.

Here, we describe the synthesis of a series of functionalized diadenosine derivatives based on the chemical structure of **NKI1** [18]. Short aminoalkyl chains were introduced at each flanking region or middle region of the diadenosine motif (Figure 2) that could be used for coupling with a NHS-activated matrix. NADK binding properties of functionalized **NKI1** were analyzed by X-ray crystallography and by measuring NADK activity, allowing selection of the candidate ligands for further covalent attachment to a matrix.

## 2. Results

### 2.1. Chemical Synthesis of Functionalized Diadenosine Derivatives

**Figure 3:**
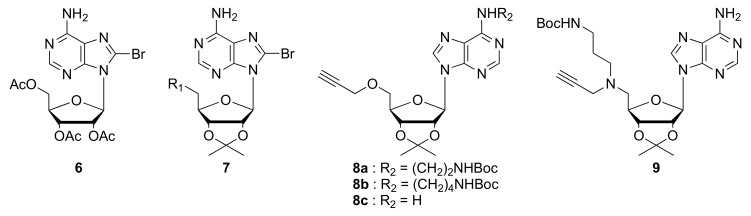
Key building blocks involved in Sonogashira cross-coupling reaction.

The key step for synthesizing derivatives **2**–**5** is a Sonogashira cross-coupling reaction between a bromide (**6** or **7**) and an alkyne (**8** or **9**) (Figure 3). The appropriate building blocks were easily prepared according to classical methodology.

**Scheme 1:**
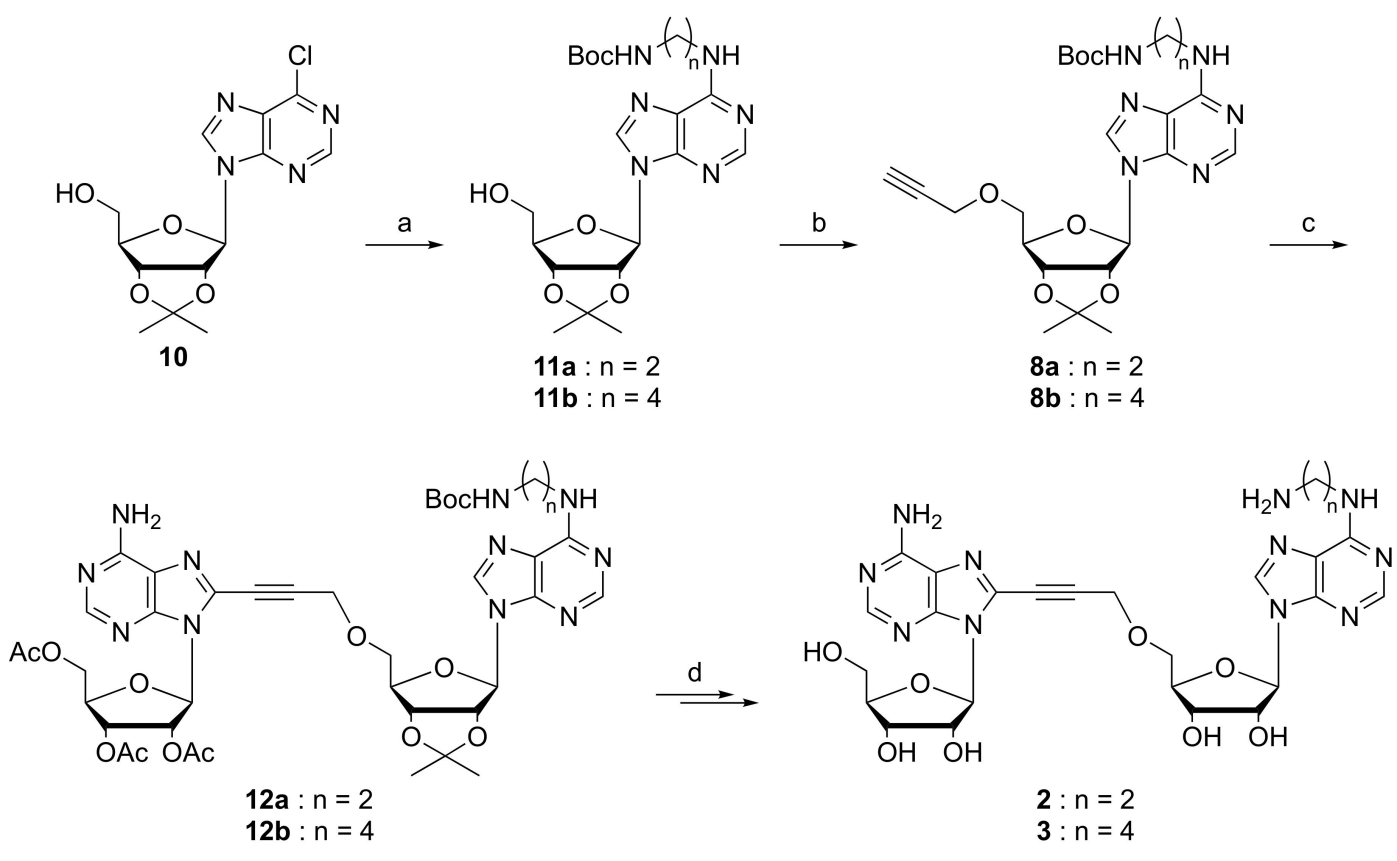
Reagents and conditions: (**a**) BocNH(CH_2_)_n_NH_2_, DME, NEt_3_, 80 °C, 66% (n = 2) or 91% (n = 4); (**b**) NaH, propargylbromide, THF, 4 °C, 60% or 68%; (**c**) **6**, Pd(PPh_3_)_4_, CuI, NEt_3_, THF, 60 °C, 37% or 40%; (**d**) 28% NH_4_OH/MeOH, rt; TFA/H_2_O, 4 °C, 42% or 61% in two steps.

The synthetic route to the N6-functionalized **NKI1** derivatives **2** and **3** is depicted in Scheme 1. An aminoalkyl side chain (2 or 4 carbon) was introduced at the 6-position of adenosine by S_N_Ar from the 6-chloropurine derivative **10**. Selective 5’-*O*-propargylation of **11a** and **11b** in the presence of propargyl bromide and NaH in DMF afforded the building blocks **8a** and **8b**, respectively, which were involved in Sonogashira cross-coupling reaction with bromide **6** to give **12a** and **12b**. Finally, removal of all protecting groups provided the desired compounds **2** and **3**.

**Scheme 2:**
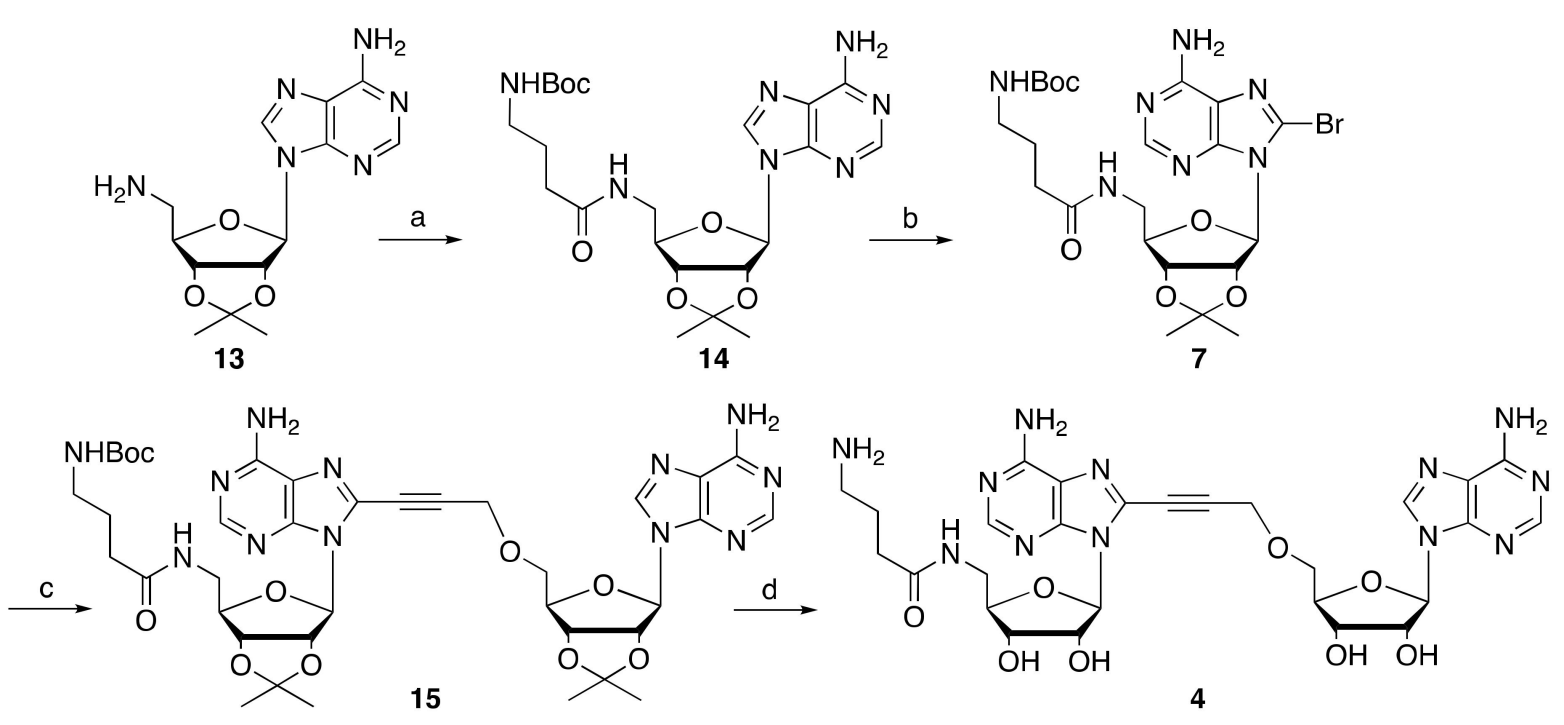
Reagents and conditions: (**a**) Boc-GABA, PyBOP, DIEA, DMF, 74%; (**b**) Br_2_, acetate buffer/dioxane, 69%; (**c**) **8c**, Pd(PPh_3_)_4_, CuI, NEt_3_, THF, 60 °C, 25%; (**d**) TFA/H_2_O, 4 °C, 45%.

The synthesis of compound **4** was achieved by reaction of 4-(*N*-Boc-amino)butyric acid (Boc-GABA-OH) with the 5’-amino derivative **13** in the presence of PyBOP and DIEA in DMF, followed by C8 bromination of the resulting amide **14** to give the key intermediate **7** (Scheme 2). Sonogashira coupling of bromide **7** and alkyne **8c** [16], followed by acidic treatment of the coupling product **15** gave the desired derivative **4** (Scheme 2).

**Scheme 3:**
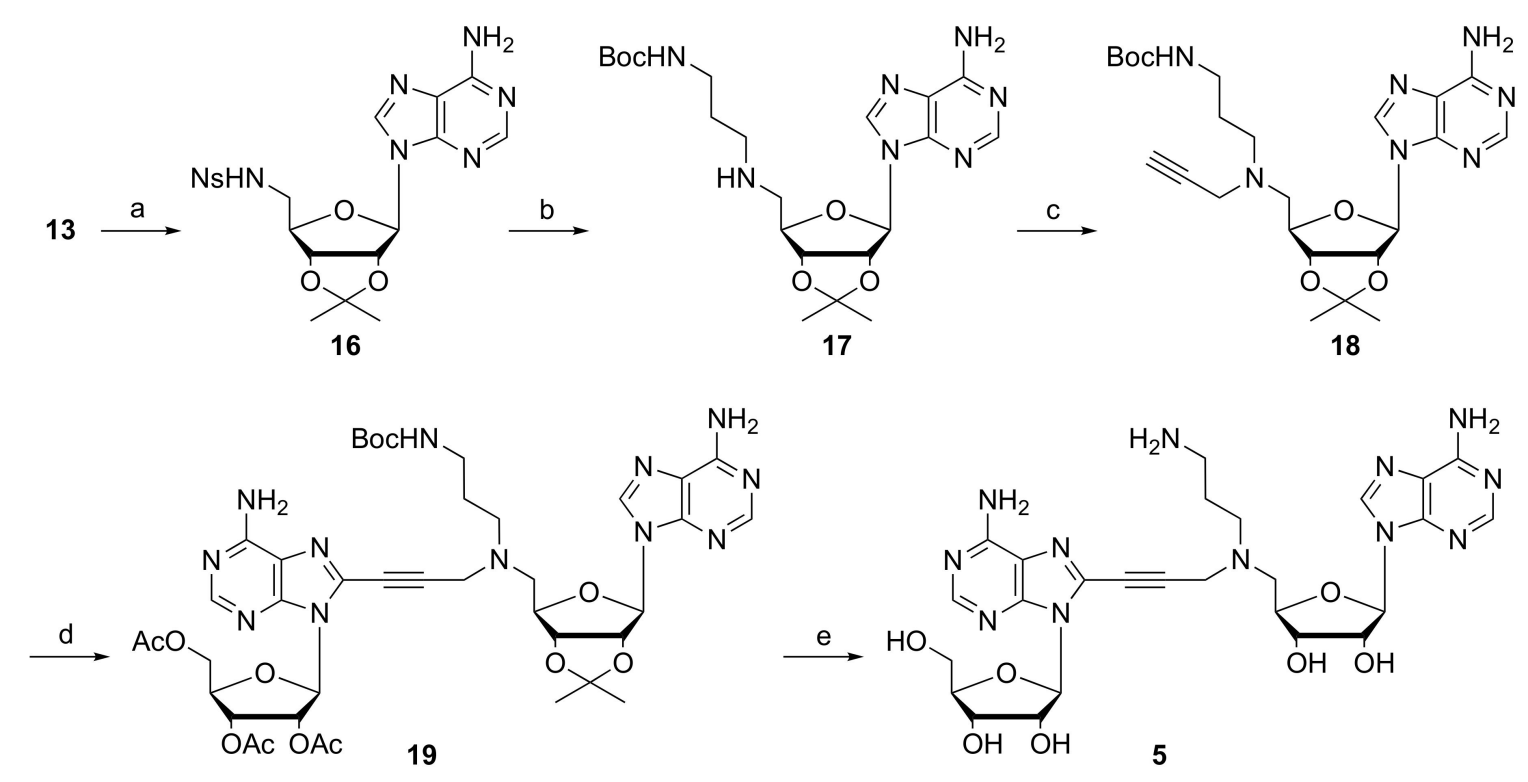
Reagents and conditions: (**a**) NsCl, pyridine, 88%; (**b**) BocNH(CH_2_)_3_Br, K_2_CO_3_, DMF, 50 °C; then PhSH, 79%; (**c**) Propargylbromide, DIEA, DMF, 82%; (**d**) **6**, Pd(PPh_3_)_4_, CuI, NEt_3_, THF, 60 °C, 63%; (**e**) 28% NH_4_OH, MeOH; TFA/H_2_O, 61% in two steps.

Finally, introduction of an aminoalkyl side chain at the middle position of the ligand was achieved from 5’-amino derivative **13** via the Ns strategy [21]. Nosylation of **13**, followed by alkylation of the *N*-sulfonamide **16** and removal of Ns group afforded the *N*-monosubstituted amine **17** (Scheme 3). Alkylation of the secondary amine using propargyl bromide afforded the key *N*-alkylated intermediate **9** in good overall yield. Sonogashira reaction between **9** and **6** led to the coupling product **19**. Finally, two-step removal of protecting groups gave the desired derivative **5**.

### 2.2. X-ray Structures of LmNADK1 Bound to Compounds **2**–**5**

**Figure 4:**
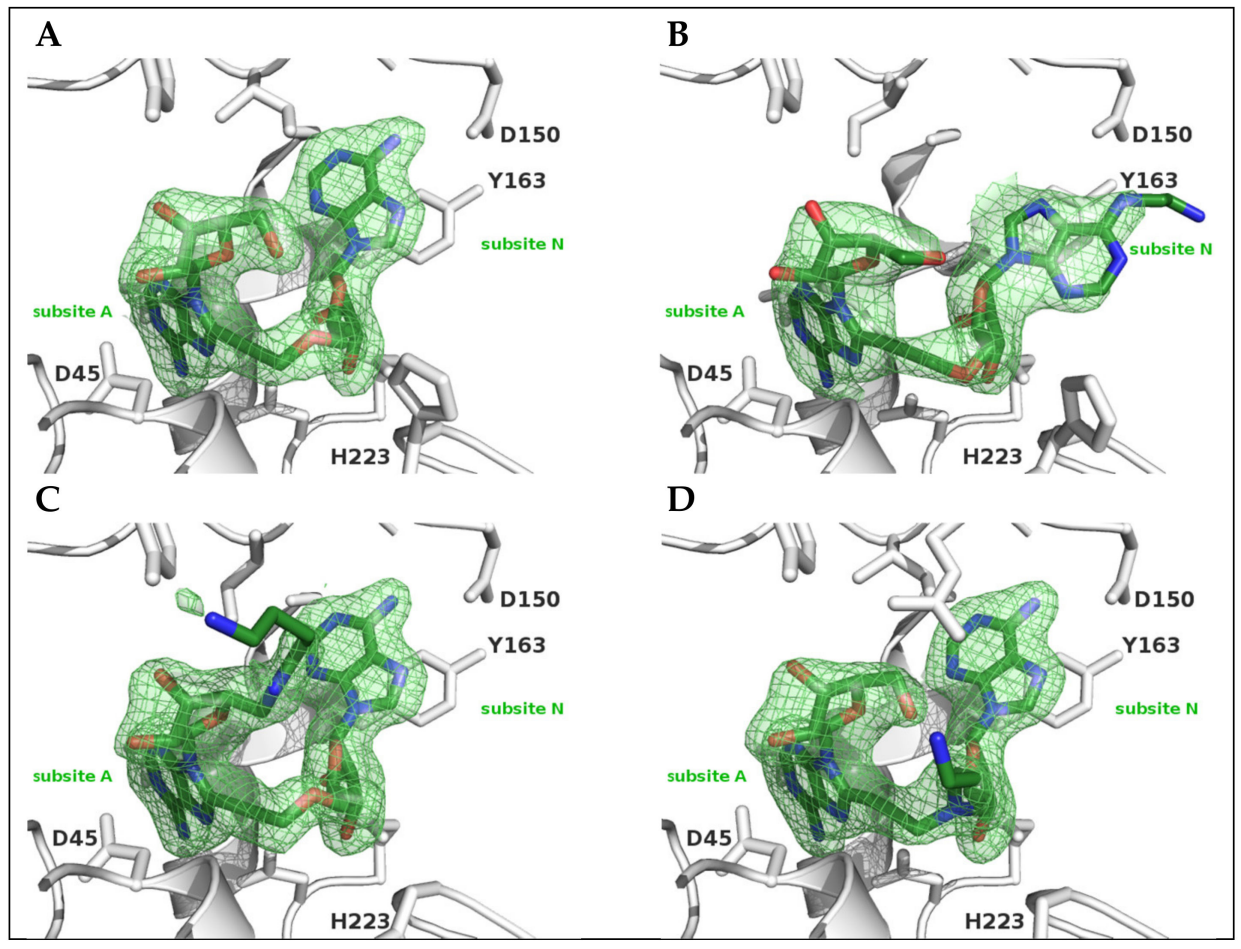
X-ray structures of *Lm*NADK1 in complex with ligands **NKI1** (6RG9), **2** (6Z61), **4** (6Z65) and **5** (6Z64). A zoomed in view of the NAD binding site is shown in the image. Important residues discussed in the text are shown in stick and labeled. Compounds **NKI1** (**A**), **2** (**B**), **4** (**C**) and **5** (**D**) are shown in sticks color according to cpk code with carbon in green. The figures were drawn using Pymol (www.pymol.org).

A prerequisite to use a chemical probe is that it retains its binding affinity for its target. The binding properties of the four synthesized derivatives **2**–**5** were analyzed by X-ray crystallography. We checked the binding properties of these new di-adenosine derivatives by determining their complex with crystallized *Lm*NADK1. The soaking procedure described previously for inhibitor 1 [18] was used to obtain crystals of the target in complex with compounds **2-5**. The X-ray structures were solved by molecular replacement using PDB6RGC [18] as a starting template and refined to 2.2–2.4 Å resolution. The resulting structures showed the same overall mode of binding to *Lm*NADK1, the ligands occupying both sub-sites A and N of the NAD binding pocket (Figure 4).

While compound **3** failed to yield complex with the crystallized target, compound **2** soaking led to a complex with the target. The structure (Figure 4B) showed a clear change in the orientation of the adenine moiety located in the subsite N (*syn vs anti* conformation), while that of the adenine moiety in the subsite A was maintained. The purine base flipping positioned the amino end out of the target, while the imidazole ring of the adenine still stacked on Tyr163. However, the N6 spacer arm appears to prevent the usual orientation of the ligand in the subsite N due to van der Waals clashes with neighboring residues, especially Asp150 which is normally hydrogen bonded to the N6 atom. The binding modes of compounds **4** and **5** were very similar to that of **NKI1**, the spacer arm having little impact on the orientation of the ligands. When the 4-carbon aminoalkyl chain is grafted at the 5’-end of the di-adenosine derivative (compound **4**), no particular interaction of this added spacer arm with any residues from the protein could be observed (Figure 4C) and the amino functional group appeared readily amenable to derivatization. Similarly, when the spacer arm was introduced between the two adenosine residues via the *N*-propargyl atom (compound **5**), the aminoalkyl chain pointed outside the NAD binding site (Figure 4D). In all three cases, the spacer arms appeared flexible in the crystal structures (weak electron densities; see Figure 4B–D) suggesting they are perfectly amenable to column grafting.

### 2.3. Effects of Compounds **2**–**5** on LmNADK1 Activity

Another important parameter to select the chemical probe is that it retains its binding properties. The potency of ligands **2**–**5** as inhibitors of recombinant *Lm*NADK1 *in vitro* were determined using the coupling assay described previously [15].

**Figure 5:**
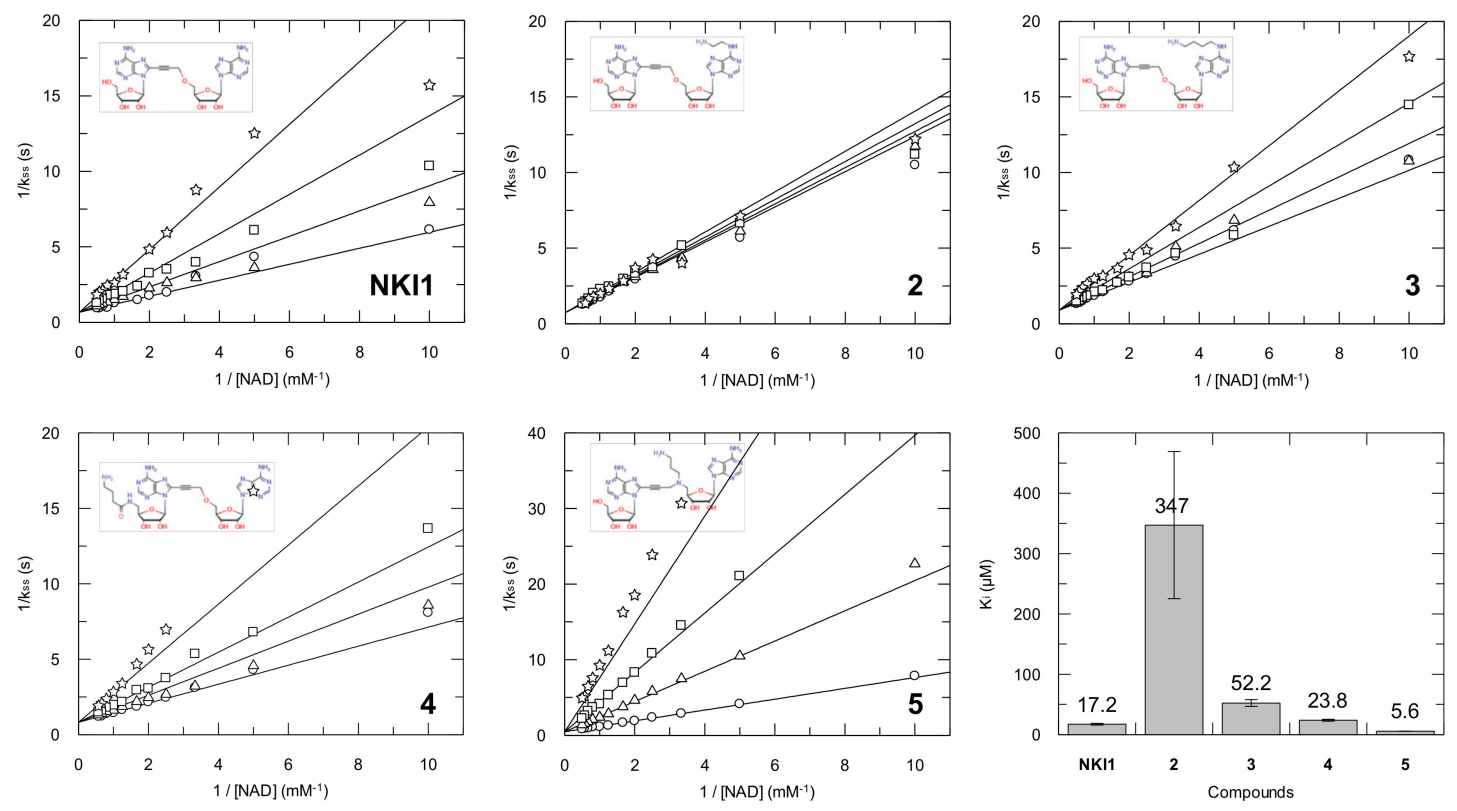
Determination of inhibition constants of compounds **1**–**5**. Inhibitor concentrations were 0 (circles), 10 (triangles), 25 (squares) and 50 (stars) µM. Secondary plots (k_ss_ as a function of initial NAD concentration) were fitted assuming a competitive inhibition using GraFit software (v 7.0.3) and are presented here as double reciprocal plots. K_i_ values determined are represented as bars (values are displayed on top of the bars).

The introduction of an aminoalkyl chain at position 6 of the adenosine residue located in the N subsite (compounds **2** and **3**) led to sharp decrease in inhibitory potency (Figure 5). This finding is consistent with our previous work describing the affinity drop induced by the introduction of a N6-cyclopropylamino group on the adenosine residue located in the subsite N [18]. Introduction of the spacer arm at the 5’-end of the diadenosine derivative (compound **4**) resulted in a slightly reduced inhibitory potency as compared to that of inhibitor **1** (**NKI1**). In contrast, when the spacer arm was attached in the middle part of the ligand, between the two adenosine residues via the *N* propargyl atom, inhibitory potency of compound **5** was slightly increased.

## 3. Discussion

By taking advantage of the substrate recognition characteristics of NADK [15,16], we designed four ligand derivatives based on the structure of our lead NADK inhibitor **NKI1** [18]. Successful synthesis of the four diadenosine derivatives bearing short aminoalkyl chains to be coupled to NHS-activated Sepharose^TM^ matrix allowed investigation of their specific properties towards NADK. NADK from *L. monocytogenes* was selected as representative target for this study, as the recombinant enzyme is easily produced and more amenable to subsequent crystallization trials than NADK from *S. aureus.* Additionally, we showed that *Sa*NADK shares 57% of sequence identity with its listerial counterpart [14,18]. While compound **3** failed to bind to *Lm*NADK, the other three derivatives yielded complexes that diffracted with 2.2–2.4 Å resolution. Compounds **4** and **5** in complex with *Lm*NADK1 showed a binding mode highly similar to that of NKI1. In contrast, compound **2** showed a different orientation which could explain its increased inhibition constant. In line with their binding properties, compounds **4** and **5** had inhibition potentials in the range of **NKI1** Ki. Compound **5** had the highest apparent affinity for the purified enzyme of all derivatives tested in our study.

Our functionalized diadenosine derivative **5**, and in a lesser extent compound **4**, have the required features to develop a specific affinity chromatography column, which could be applied to bacterial NADKs. Ultimately, this chemical probe will help to decipher the selectivity characteristics of our lead compound.

## 4. Materials and Methods

### 4.1. General Information

Commercially available reagents and solvents, unless otherwise stated, were used without purification. Anhydrous reactions were carried out under an argon atmosphere. Analytical thin-layer chromatography (TLC) was performed on TLC plates pre-coated with silica gel 60 F_254_. Compounds were visualized with UV light (254 nm) and by spraying with a mixture of ethanol/anisaldehyde/sulfuric acid/acetic acid (90/5/4/1), followed by heating. Reactions were also monitored using an HPLC system (Agilent (Agilent Technologies, France, Les Ulis) 1100 equipped with a C18 reverse phase column) coupled to a mass spectrometer (ESI source). Flash chromatography was performed with silica gel 60 (230–400 mesh). HPLC purification was carried out on an Agilent system (1100 Series) equipped with a diode array detector using a C18 reverse phase column (Kromasil, 5 μm, 100 Å, 150 × 10 mm) and a linear gradient of acetonitrile in 10 mM triethylammonium acetate (TEAA) buffer over 15 or 20 min at a flow rate of 4 mL/min. ^1^H and ^13^C NMR spectra were recorded on Bruker Avance 400 (Bruker, France, Wissembourg) at 400.13 MHz and 100.62 MHz, respectively. Chemical shifts (*δ*) are reported in parts per million (ppm) relative to the solvent signals. Coupling constants (*J* values) are reported in Hz. The complete assignment of ^1^H and ^13^C signals was performed by analysis of the correlated homonuclear ^1^H,{^1^H}–COSY and heteronuclear ^1^H,{^13^C}–HMBC, ^1^H,{^13^C}–HSQC spectra (see copies of ^1^H and ^13^C NMR spectra of prepared compounds in the Supplementary Materials). High-resolution mass spectra (HRMS) were recorded with a Q-Tof Micro mass spectrometer under electrospray ionization (ESI) using 0.1% formic acid in acetonitrile/water in positive ion mode. The purity of all tested compounds was greater than 97% (HPLC analysis). Retention time (*t_R_*) and gradient are specified.

### 4.2. Synthesis

*2′,3′,5′-Tri-O-acetyl-8-bromoadenosine (**6**).* The title compound was obtained by peracetylation of commercially available 8-bromoadenosine (90% yield).

*6-Chloro-9-(2′,3′-O-isopropylidene-ß-D-ribofuranosyl)purine (**10**).* The title compound was obtained by reaction of 6-chloropurine riboside with 2,2-dimethoxypropane in acetone in the presence of toluene-4-sulfonic acid for 3 h at room temperature (91% yield) [22].

*N^6^-(2-tert-Butyloxycarbonyl)aminoethyl-2’,3’-O-isopropylidene-adenosine (**11a**).* To a stirred solution of 6-chloro-9-(2′,3′-*O*-isopropylidene-*ß*-d-ribofuranosyl)purine (**10**) (0.98 g, 3.0 mmol) in anhydrous DME (15 mL) was added NEt_3_ (1.25 mL, 9.0 mmol), followed by a solution of *N-*Boc-ethylenediamine (prepared according to reported procedures) [23] (1.44 g, 9.0 mmol) in anhydrous DME (15 mL). The stirred mixture was heated at 80 °C overnight under an argon atmosphere, then concentrated under reduced pressure. The residue was dissolved in dichloromethane (80 mL), washed in turn with water (80 mL) and brine (80 mL). The organic layer was dried over Na_2_SO_4_ and concentrated under reduced pressure. The crude product was purified by flash column chromatography (50 g SiO_2_, 0 to 3% methanol in dichloromethane) to afford compound **11a** (0.90 g, 66%) as a white foam. ^1^H NMR (400 MHz, CDCl_3_): *δ* 1.40 (s, 3H, CH_3_ isop), 1.44 (s, 9H, CH_3_ Boc), 1.66 (s, 3H, CH_3_ isop), 3.44 -3.48 (m, 2H, ***CH_2_***NHBoc), 3.76-3.84 (m, 3H, ***CH_2_***NH-6 and H-5’), 3.99 (dd, *J* = 1.4 Hz, *J* = 12.8 Hz, 1H, H-5”), 4.53-4.56 (m, 1H, H-4’), 5.09 (br s, 1H, NHBoc), 5.13 (dd, *J* = 5.8 Hz, *J* = 1.0 Hz, 1H, H-3’), 5.21 (pt, *J* = 5.5 Hz, 1H, H-2’), 5.87 (d, *J* = 4.9 Hz, 1H, H-1’), 6.40-6.90 (br s, 2H, NH-6 and OH-5’), 7.84 (s, 1H, H-8), 8.33 (s, 1H, H-2); ^13^C NMR (100 MHz, CDCl_3_): *δ* 25.25 (CH_3_ isop), 27.66 (CH_3_ isop), 28.34 (3 CH_3_ Boc), 40.74 (***CH_2_***NH-6 and ***CH_2_***NHBoc), 63.42 (C-5’), 79.59 (Cq Boc), 81.71 (C-3’), 83.09 (C-2’), 86.15 (C-4’), 94.32 (C-1’), 113.98 (Cq isop), 121.42 (C-5), 139.76 (C-8), 147.61 (C-4), 152.91 (C-2), 155.35 (C-6), 156.30 (CO Boc); HRMS (ESI-TOF): *m/z* calcd for [C_20_H_30_N_6_O_6_+H]^+^ 451.2305, found 451.2293.

*N^6^-(2-tert-Butyloxycarbonyl)aminobutyl-2’,3’-O-isopropylidene-adenosine (**11b**).* Compound **11b** was prepared from **10** (0.98 g, 3.0 mmol) and *N-*Boc-1,4-butanediamine (prepared according to reported procedures) [23] (1.69 g, 9.0 mmol) following the same procedure as for **11a**. Purification by flash column chromatography (50 g SiO_2_, 0 to 3% methanol in dichloromethane) yielded compound **11b** (1.31 g, 91%) as a white foam. ^1^H NMR (400 MHz, CDCl_3_): *δ* 1.39 (s, 3H, CH_3_ isop), 1.45 (s, 9H, CH_3_ Boc), 1.61 (quint, *J* = 7.0 Hz, 2H, CH_2_), 1.66 (s, 3H, CH_3_ isop), 1.74 (quint, *J* = 7.0 Hz, 2H, CH_2_), 3.19 (br q, 2H, ***CH_2_***NHBoc), 3.68 (br s, 2H, ***CH_2_***NH-6), 3.80 (br d, *J* = 12.8 Hz, 1H, H-5’), 3.99 (dd, *J* = 1.3 Hz, *J* = 12.8 Hz, 1H, H-5”), 4.53-4.56 (m, 1H, H-4’), 4.78 (br s, 1H, NHBoc), 5.13 (dd, *J* = 1.0 Hz, *J* = 5.9 Hz, 1H, H-3’), 5.22 (pt, *J* = 5.8 Hz, 1H, H-2’), 5.86 (d, *J* = 4.8 Hz, 1H, H-1’), 6.04 (br s, 1H, OH-5’), 6.67 (br s, 1H, NH-6), 7.79 (s, 1H, H-8), 8.33 (s, 1H, H-2); ^13^C NMR (100 MHz, CDCl_3_): *δ* 25.34 (CH_3_ isop), 26.97 (CH_2_), 27.24 (CH_2_), 27.65 (CH_3_ isop), 28.42 (CH_3_ Boc), 40.16 (***CH_2_***NH-6 and ***CH_2_***NHBoc), 63.42 (C-5’), 79.15 (C_q_ Boc), 81.72 (C-3’), 83.06 (C-2’), 86.14 (C-4’), 94.35 (C-1’), 113.94 (C_q_ isop), 121.25 (C-5), 139.52 (C-8), 147.31 (C-4), 152.69 (C-2), 155.25 (C-6), 156.01 (CO Boc); HRMS (ESI-TOF): *m/z* calcd for [C_22_H_34_N_6_O_6_+H]^+^ 479.2618, found 479.2614.

*N6-(2-tert-Butyloxycarbonyl)aminoethyl-2’,3’-O-isopropylidene-5’-O-propargyl-adenosine (**8a**).* To a stirred suspension of sodium hydride (0.10 g 60% in oil, 2.5 mmol) in anhydrous THF (12 mL) was added at 0 °C a solution of **11a** (0.56 g, 1.2 mmol) in THF (12 mL). The reaction mixture was stirred at 0 °C for 1 h, then propargyl bromide (80% in toluene, 0.13 mL, 1.2 mmol) was added dropwise. After 24 h at 4 °C, the reaction was quenched by adding glacial acetic acid (0.14 mL) and stirring for 1 h at 4 °C. After removal of the volatiles under reduced pressure, the crude residue was dissolved ethyl acetate (50 mL) and washed twice with water (60 mL). The organic layer was dried, concentrated under reduced pressure and the residue purified by flash column chromatography (30 g SiO_2_, 0 to 3% methanol in dichloromethane) affording compound **8a** (0.35 g, 60%) as a white foam. ^1^H NMR (400 MHz, CDCl_3_): *δ* 1.41 (s, 3H, CH_3_ isop), 1.43 (s, 9H, CH_3_ Boc), 1.64 (s, 3H, CH_3_ isop), 2.44 (t, *J* = 2.3 Hz, 1H, HC≡), 3.44 (br q, *J* = 5.6 Hz, 2H, ***CH_2_***NHBoc), 3.73 (dd, *J* = 4.8 Hz, *J* = 10.3 Hz, 1H, H-5’), 3.78 (dd, *J* = 3.9 Hz, *J* = 10.3 Hz, 1H, H-5”), 3.81-3.85 (m, 2H, ***CH_2_***NH-6), 4.14 (d, *J* = 2.3 Hz, 2H, ***CH_2_***C≡), 4.49-4.53 (m, 1H, H-4’), 5.03 (dd, *J* = 2.7 Hz, *J* = 6.2 Hz, 1H, H-3’), 5.17 (br s, 1H, NHBoc), 5.34 (dd, *J* = 2.3 Hz, *J* = 6.2 Hz, 1H, H-2’), 6.19 (d, *J* = 2.4 Hz, 1H, H-1’), 6.33 (br s, 1H, NH-6), 8.01 (s, 1H, H-8), 8.39 (s, 1H, H-2); ^13^C NMR (100 MHz, CDCl_3_): *δ* 25.38 (CH_3_ isop), 27.18 (CH_3_ isop), 28.35 (3 CH_3_ Boc), 40.90 (***CH_2_***NH-6 and ***CH_2_***NHBoc), 58.57 (C≡C-***CH_2_***), 69.85 (C-5’), 75.24 (***C***≡C-CH_2_), 78.80 (C≡***C***-CH_2_), 79.44 (C_q_ Boc), 81.88 (C-3’), 84.76 (C-2’), 85.74 (C-4’), 91.20 (C-1’), 114.23 (C_q_ isop), 120.16 (C-5), 138.93 (C-8), 148.87 (C-4), 152.91 (C-2), 154.88 (C-6), 156.27 (CO Boc); HRMS (ESI-TOF): *m/z* calcd for [C_23_H_32_N_6_O_6_+H]^+^ 489.2462, found 489.2463.

*N^6^-(2-tert-Butyloxycarbonyl)aminobutyl-2’,3’-O-isopropylidene-5’-O-propargyl-adenosine **(8b)**.* Compound **8b** was prepared from **11b** (0.95 g, 2.0 mmol) following the same procedure as for **8a**. Purification by flash column chromatography (70 g SiO_2_, 0 to 2% methanol in dichloromethane) afforded compound **8b** (0.70 g, 68%). ^1^H NMR (400 MHz, CDCl_3_): *δ* 1.36 (s, 3H, CH_3_ isop), 1.41 (s, 9H, CH_3_ Boc), 1.51-1.63 (m, 2H, CH_2_), 1.59 (s, 3H, CH_3_ isop), 1.69 (quint, *J* = 7.0 Hz, 2H, CH_2_), 2.42 (t, *J* = 2.3 Hz, 1H, HC≡), 3.10-3.19 (m, 2H, ***CH_2_***NHBoc), 3.60-3.66 (m, 2H, ***CH_2_***NH-6), 3.69 (dd, *J* = 4.9 Hz, *J* = 10.3 Hz, 1H, H-5’), 3.74 (dd, *J* = 4.0 Hz, *J* = 10.3 Hz, 1H, H-5”), 4.10 (d, *J* = 2.3 Hz, 2H, ***CH_2_***C≡), 4.44-4.49 (m, 1H, H-4’), 4.99 (dd, *J* = 2.6 Hz, *J* = 6.1 Hz, 1H, H-3’), 5.32 (dd, *J* = 2.3 Hz, *J* = 6.3 Hz, 1H, H-2’), 6.05 (br s, 1H, NH-6), 6.15 (d, *J* = 2.3 Hz, 1H, H-1’), 7.94 (s, 1H, H-8), 8.34 (s, 1H, H-2); ^13^C NMR (100 MHz, CDCl_3_): *δ* 25.34 (CH_3_ isop), 27.08 (CH_3_ isop), 27.14 and 27.19 (2 CH_2_), 28.40 (3 CH_3_ Boc), 40.12 (***CH_2_***NH-6 and ***CH_2_***NHBoc), 58.53 (C≡C-***CH_2_***), 69.82 (C-5’), 75.30 (***C***≡C-CH_2_), 78.83 (C≡***C***-CH_2_), 78.99 (C_q_ Boc), 81.85 (C-3’), 84.68 (C-2’), 85.68 (C-4’), 91.06 (C-1’), 114.17 (C_q_ isop), 120.07 (C-5), 138.66 (C-8), 148.68 (C-4), 153.23 (C-2), 154.94 (C-6), 156.04 (CO Boc); HRMS (ESI-TOF): *m/z* calcd for [C_25_H_36_N_6_O_6_+H]^+^ 517.2775, found 517.2780.

*2’,3’,5’-Tri-O-acetyl-8-[3-(N^6^-(2-tert-butyloxycarbonyl)aminoethyl-2’,3’-O-isopropylidene-5’-O-adenosinyl)propargyl]adenosine **(12a)**.* To an argon-degassed solution (three times) of 8a (0.20 g, 0.42 mmol) and bromide 6 (0.31 g, 0.63 mmol) in anhydrous THF (8 mL) in the presence of NEt_3_ (0.18 mL, 1.26 mmol) were added CuI (8 mg, 0.042 mmol) and Pd(PPh_3_)_4_ (24 mg, 0.021 mmol). The stirred mixture was degassed 3 times, then heated at 60 °C under argon atmosphere for 5 h. The volatiles were removed under reduced pressure and the residue purified by flash column chromatography (20 g SiO_2_, 0 to 4% methanol in dichloromethane) affording compound **12a** (0.15 g, 40%) and the homocoupling product (63 mg, 8%). ^1^HNMR (400 MHz, CDCl_3_): δ 1.41 (s, 3H, CH_3_ isop), 1.43 (s, 9H, CH_3_ Boc), 1.64 (s, 3H, CH_3_ isop), 2.05 (s, 3H, COCH_3_), 2.10 (s, 3H, COCH_3_), 2.14 (s, 3H, COCH_3_), 3.39-3.47 (m, 2H, CH_2_NHBoc) 3.77 (br, 2H, CH_2_NH-6), 3.84 (dd, J = 5.1 Hz, J = 10.2 Hz, 1H, H-5’B), 3.92 (dd, J = 4.0 Hz, J = 10.2 Hz, 1H, H-5”B), 4.32 (dd, J = 6.0 Hz, J = 11.8 Hz, 1H, H-5’A), 4.41 (td, J = 6.0 Hz, J = 11.8 Hz, 1H, H-4’A), 4.47 (br, 2H, CH_2_C≡), 4.50-4.55 (m, 2H, H-4’B and H-5”A), 5.07 (dd, J = 3.0 Hz, J = 6.2 Hz, 1H, H-3’B), 5.23 (br s, 1H, NHBoc), 5.35-5.41 (br s, 1H, H-2’B), 5.96 (t, J = 6.1 Hz, 1H, H-3’A), 6.19 (d, J = 2.2 Hz, 1H, H-1’B), 6.22 (d, J = 4.2 Hz, 1H, H-1’A), 6.24 (br s, 2H, NH_2_), 6.30 (dd, J = 4.2 Hz, J = 6.1 Hz, 1H, H-2’A), 6.46 (br s, 1H, NH-6B), 8.02 (s, 1H, H-8B), 8.37 (s, 1H, H-2A), 8.42 (s, 1H, H-2B); ^13^CNMR (100 MHz, CDCl_3_): δ 20.43 (COCH_3_), 20.49 (COCH_3_), 20.64 (COCH_3_), 25.36 (CH_3_ isop), 27.16 (CH_3_ isop), 28.36 (3 CH_3_ Boc), 40.71 (CH_2_NHBoc and CH_2_NH-6), 59.04 (C≡C-CH_2_), 63.05 (C-5’A), 70.49 (C-5’B), 70.54 (C-3’A), 72.52 (C-2’A), 76.69 (C≡C-CH_2_), 79.49 (C_q_ Boc), 79.86 (C-4’A), 81.80 (C-3’B), 84.70 (C-2’B), 85.74 (C-4’B), 87.37 (C-1’A), 91.12 (C-1’B), 91.80 (C≡C-CH_2_), 114.36 (C_q_ isop), 119.78 (C-5A), 120.20 (C-5B), 133.26 (C-8A), 138.88 (C-8B); 148.90 (C-4B); 149.50 (C-4A), 153.23 (C-2B), 154.26 (C-2A), 155.00 (C-6B), 155.52 (C-6A), 156.31 (CO Boc), 169.42 (CO), 169.50 (CO), 170.58 (CO); HRMS (ESI-TOF): m/z calcd for [C_39_H_49_N_11_O_13_+H]^+^ 880.3589, found 880.3582.

*2’,3’,5’-Tri-O-acetyl-8-[3-(N^6^-(4-tert-butyloxycarbonyl)aminobutyl-2’,3’-O-isopropylidene-5’-O-adenosinyl)propargyl]adenosine **(12b).*** Compound **12b** was prepared from **8b** (206 mg, 0.4 mmol) and bromide **6** (184 mg, 0.26 mmol) following the same procedure as for **12a**. Purification by chromatography on silica gel (1 to 4% methanol in dichloromethane) gave compound 12b (88 mg, 37%). ^1^H NMR (400 MHz, CDCl_3_): δ 1.41 (s, 3H, CH_3_ isop), 1.46 (s, 9H, CH_3_ Boc), 1.56-1.66 (m, 2H, CH_2_), 1.64 (s, 3H, CH_3_ isop), 1.67-1.79 (m, 2H, CH_2_), 2.05 (s, 3H, COCH_3_), 2.10 (s, 3H, COCH_3_), 2.15 (s, 3H, COCH_3_), 3.15-3.25 (m, 2H, CH_2_NHBoc), 3.60-3.70 (m, 2H, CH_2_NH-6), 3.85 (dd, J = 5.2 Hz, J = 10.1 Hz, 1H, H-5’B), 3.92 (dd, J = 4.2 Hz, J = 10.1 Hz, 1H, H-5”B), 4.33 (dd, 1H, J = 6.0 Hz, J = 11.8 Hz, H-5”A), 4.38-4.44 (m, 1H, H-4”A), 4.48 (d, J = 1.0 Hz, 2H, CH_2_C≡), 4.50-4.55 (m, 2H, H-4’B and H-5”A), 5.10 (dd, J = 3.0 Hz, J = 6.2 Hz, 1H, H-3’B), 5.41 (dd, J = 2.2 Hz, J = 6.2 Hz, 1H, H-2’B), 5.93 (br, 2H, NH_2_), 5.96 (pt, 1H, J = 6.0 Hz, H-3’A), 6.18 (d, J = 2.2 Hz, 1H, H-1’B), 6.23 (d, 1H, J = 4.2 Hz, H-1’A), 6.30 (dd, J = 4.2 Hz, J = 6.0 Hz, 1H, H-2’A), 7.96 (s, 1H, H-8B), 8.39 (s, 1H, H-2A), 8.43 (s, 1H, H-2B); ^13^C NMR (100 MHz, CDCl_3_): δ 20.44 (COCH_3_), 20.51 (COCH_3_), 20.65 (COCH_3_), 25.36 (CH_3_ isop), 27.04 (CH_2_), 27.16 (CH_3_ isop), 27.26 (CH_2_), 28.44 (3 CH_3_ Boc), 40.03 (CH_2_NH-6 and CH_2_NHBoc), 59.05 (C≡C-CH_2_), 63.05 (C-5’A), 70.50 (C-3’A), 70.56 (C-5’B), 72.50 (C-2’A), 75.62 (C≡C-CH_2_), 79.16 (Cq Boc), 79.88 (C-4’A), 81.86 (C-3’B), 84.59 (C-2’B), 85.79 (C-4’B), 87.41 (C-1’A), 91.05 (C-1’B), 91.91 (C≡C-CH_2_), 114.37 (Cq isop), 118.13 (C-5A); 119.80 (C-5B), 133.42 (C-8A), 138.71 (C-8B), 148.92 (C-4B); 149.40 (C-4A); 153.43 (C-2B); 154.27 (C-2A); 154.96 (C-6B), 155.38 (C-6A), 156.05 (CO Boc), 169.37 (CO), 169.48 (CO), 170.55 (CO); HRMS (ESI-TOF): m/z calcd for [C_41_H_53_N_11_O_13_+H]^+^ 908.3803, found 908.4268.

*8-[3-(N^6^-2-Aminoethyl-5’-O-adenosinyl)propargyl]adenosine **(2)**.* To a solution of **12a** (46 mg, 0.05 mmol) in MeOH (5 mL) was added 28% NH_4_OH (1 mL). After stirring overnight at room temperature, the reaction mixture was concentrated under reduced pressure, and the crude product (37 mg) was used in the next step without further purification. To the foam (37 mg, 0.05 mmol) was added at 0 °C a 70% aqueous solution of TFA (1 mL). After stirring for 2 h, water was added and the mixture was lyophilized. Purification by reverse phase HPLC (0 to 20% acetonitrile in 10 mM TEAA buffer over 15 min, *t*_R_ = 13.3 min) afforded compound **2** as acetate salt (13 mg, 42% in two steps). ^1^H NMR (400 MHz, DMSO-*d*_6_): *δ* 1.88 (s, 1.3H, CH_3_ Ac), 2.81-2.86 (m, 2H, ***CH_2_***NH_2_), 3.54 (d, *J* = 4.2 Hz, *J* = 12.2 Hz, 1H, H-5’A), 3.59 (br, 2H, ***CH_2_***NH-6), 3.68 (d, *J* = 3.9 Hz, *J* = 12.2 Hz, 1H, H-5’’A), 3.76 (d, *J* = 5.5 Hz, *J* = 10.6 Hz, 1H, H-5’B), 3.85 (dd, *J* = 3.6 Hz, *J* = 10.6 Hz, 1H, H-5”B), 3.97-4.02 (m, 1H, H-4’A), 4.08-4.13 (m, 1H, H-4’B), 4.19-4.23 (m, 2H, H-3’A and H-3’B), 4.60 (s, 2H, ***CH_2_***C≡), 4.63 (t, 1H, *J* = 5.1 Hz, H-2’B), 5.01 (dd, *J* = 5.3 Hz, *J* = 6.6 Hz, 1H, H-2’A), 5.93 (d, 1H, *J* = 5.1 Hz, H-1’B), 5.95 (d, *J* = 6.6 Hz, 1H, H-1’A), 7.63 (br s, 2H, NH_2_), 7.73 (br s, 2H, NH_2_), 8.17 (s, 1H, H-2B), 8.24 (s, 1H, H-2A), 8.33 (s, 1H, H-8B); ^13^C NMR (100 MHz, DMSO-*d*_6_): *δ* 21.83 (CH_3_ Ac), 40.94 (***CH_2_***NH_2_ and ***CH_2_***NH-6), 58.76 (C≡C-***CH_2_***), 62.63 (C-5’A), 70.74 (C-5’B), 71.00 (C-3’B), 71.43 (C-3’A), 72.11 (C-2’A), 73.71 (C-2’B), 75.55 (***C***≡C-CH_2_), 83.33 (C-4’B), 87.16 (C-4’A), 87.98 (C-1’B), 89.92 (C-1’A), 92.60 (C≡***C***-CH_2_), 119.83 (C-5A and C-5B), 133.45 (C-8A), 139.76 (C-8B), 148.89 (C-4A and C-4B), 153.07 (C-2A), 153.90 (C-2B), 156.63 (C-6A and C-6B), 172.70 (CO Ac); HRMS (ESI-TOF): *m/z* calcd for [C_25_H_31_N_11_O_8_+H]^+^ 614.2435, found 614.2433.

*8-[3-(N^6^-4-Aminobutyl-5’-O-adenosinyl)propargyl]adenosine **(3).*** Compound **3** was obtained from **12b** (63 mg, 0.07 mmol) following the same procedure as for **2**. Purification by reverse phase HPLC (0 to 30% acetonitrile in 10 mM TEAA buffer over 20 min, *t*_R_ = 8.7 min) afforded compound **3** as acetate salt (22.6 mg, 50% in two steps). ^1^H NMR (400 MHz, DMSO-*d*_6_): *δ* 1.48-1.58 (m, 2H, CH_2_), 1.58-1.69 (m, 2H, CH_2_), 1.84 (s, 1H, CH_3_ Ac), 2.73 (t, *J =* 7.2 Hz, 2H, ***CH_2_***NH_2_), 3.50 (br, 2H, ***CH_2_***NH-6), 3.54 (dd, *J* = 4.2 Hz, *J* = 12.1 Hz, 1H, H-5’A), 3.69 (m, *J* = 3.9 Hz, *J* = 12.2 Hz, 1H, H-5”A), 3.76 (dd, *J* = 5.5 Hz, *J* = 10.5 Hz, 1H, H-5’B), 3.85 (dd, *J* = 3.8 Hz, *J* = 10.5 Hz, 1H, H-5”B), 3.98-4.01 (m, 1H, H-4’A), 4.08-4.12 (m, 1H, H-4’B), 4.19-4.23 (m, 2H, H-3’A and H-3’B), 4.60 (s, 2H, CH_2_C≡), 4.63 (pt, *J* = 5.3 Hz, 1H, H-2’B), 5.00 (dd, *J* = 5.3 Hz, *J* = 6.7 Hz, 1H, H-2’A), 5.93 (d, *J* = 5.4 Hz, 1H, H-1’B), 5.95 (d, *J* = 6.7 Hz, 1H, H-1’A), 7.63 (br s, 2H, NH_2_), 7.79 (br s, 2H, NH_2_), 8.17 (s, 1H, H-2B), 8.23 (s, 1H, H-2A), 8.32 (s, 1H, H-8B); ^13^C NMR (100 MHz, DMSO-*d_6_*): *δ* 23.40 (CH_3_ Ac), 25.81 (CH_2_), 26.53 (CH_2_), 39.39 (***CH_2_***NH_2_ and ***CH_2_***NH-6), 58.73 (C≡C-***CH_2_***), 62.49 (C-5’A), 70.66 (C-5’B), 70.83 (C-3’B), 71.29 (C-3’A), 72.12 (C-2’A), 73.56 (C-2’B), 75.51 (***C***≡C-CH_2_), 83.33 (C-4’B), 87.07 (C-4’A), 87.82 (C-1’B), 89.88 (C-1’A), 92.72 (C≡***C***-CH_2_), 119.71 (C-5A and C-5B), 133.45 (C-8A), 139.76 (C-8B), 148.83 (C-4A and C-4B), 153.18 (C-2A), 153.88 (C-2B), 156.40 (C-6A and C-6B), 173.90 (CO Ac); HRMS (ESI-TOF): *m/z* calcd for [C_27_H_35_N_11_O_8_ + H]^+^ 642.2748, found 642.2748.

*5’-[4-(N-tert-Butyloxycarbonyl-amino)butylamido]-5’-deoxy-2’,3’-O-isopropylidene-adenosine **(14)*****.** To a solution of 5’-amino-5’-deoxy-2’,3’-*O*-isopropylidene-adenosine (**13**) **[24]** (0.31 g, 1.0 mmol) in DMF were added DIEA (0.35 mL, 2.0 mmol), PyBOP (0.52 g, 1.0 mmol) and 4-(*tert*-butoxycarbonylamino)-butyric acid (0.20 g, 1.0 mmol). After stirring for 1 h at room temperature, the reaction was diluted with water (40 mL) and extracted with ethyl acetate (2x 80 mL). The combined organic layers were dried over Na_2_SO_4_, filtered and concentrated under reduced pressure. The residue was purified by flash column chromatography (18 g SiO_2_, 0 to 5% methanol in dichloromethane) to yield compound **3** (0.36 g, 74%) as a white foam. ^1^H NMR (400 MHz, DMSO-*d_6_*): *δ* 1.32 (s, 3H, CH_3_ isop), 1.36 (s, 9H, CH_3_ Boc), 1.54 (s, 3H, CH_3_ isop), 1.59 (quint, *J* = 7.2 Hz, 2H, CH_2_), 2.09 (td, *J* = 2.4 Hz, *J* = 7.2 Hz, 2H, ***CH_2_***CO), 2.89 (br q, *J* = 6.6 Hz, 2H, ***CH_2_***NHBoc), 3.30-3.35 (m, 2H, H-5’ and H5’’), 4.17 (td, *J* = 3.2 Hz, *J* = 5.7 Hz, 1H, H-4’), 4.90 (dd, *J* = 6.3 Hz, *J* = 3.2 Hz, 1H, H-3’), 5.43 (dd, *J* = 3.0 Hz, *J* = 6.3 Hz, 1H, H-2’), 6.12 (d, *J* = 3.0 Hz, 1H, H-1’), 6.75 (br s, 1H, NHBoc), 7.34 (s, 2H, NH_2_) 8.06 (br, 1H, NHCO), 8.19 (s, 1H, H-2), 8.32 (s, 1H, H-8); ^13^C NMR (100 MHz, DMSO-*d_6_*): *δ* 25.70 (CH_3_ isop), 26.15 (CH_2_), 27.50 (CH_3_ isop), 28.70 (3 CH_3_ Boc), 33.14 (***CH_2_***CO), 39.95 (***CH_2_***NHBoc), 41.16 (C-5’), 77.85 (Cq Boc), 82,17 (C-3’), 83.30 (C-2’), 84.63 (C-4’), 89.55 (C-1’), 113.99 (Cq isop), 119.80 (C-5), 140.53 (C-8), 149.25 (C-4), 153.19 (C-2), 156.02 (CO Boc), 156.67 (C-6), 172.68 (CONH); HRMS (ESI-TOF): *m/z* calcd for [C_22_H_33_N_7_O_6_+H]^+^ 492.2570, found 492.2582.

*8-Bromo-5’-(4-(N-tert-butyloxycarbonyl-amino)butylamido)-5’-deoxy-2’,3’-O-isopropylidene-adenosine **(7)**.* To a solution of **14** (0.17 g, 0.35 mmol) in 1,4-dioxane (1.4 mL) and sodium acetate buffer (2.1 mL, 0.5 M, pH = 5.3) was added bromine (36 µL, 0.69 mmol) dropwise. After stirring for 3 h at room temperature, the reaction was quenched by adding a saturated aqueous solution of Na_2_S_2_O_3_ (10 mL). After full discoloration, ethyl acetate (80 mL) was added and the organic layer was washed with water (20 mL) and brine (20 mL). The organic layer was dried over Na_2_SO_4_, filtered and concentrated under reduced pressure. The residue was purified by flash column chromatography (10 g SiO_2_, 0-4% methanol in dichloromethane) to yield compound **7** (0.135 g, 69%) as a pale yellow foam. ^1^H NMR (400 MHz, CDCl_3_): *δ* 1.38 (s, 3H, CH_3_ isop), 1.42 (s, 9H, CH_3_ Boc), 1.65 (s, 3H, CH_3_ isop), 1.83-1.92 (m, 2H, CH_2_), 2.32-2.40 (m, 2H, ***CH_2_***CO), 3.18-3.23 (m, 2H, ***CH_2_***NHBoc), 3.37 (dt, *J* = 3.0 Hz, *J* = 14.3 Hz, 1H, H-5’), 3.99 (ddd, *J* = 4.0 Hz, *J* = 8.0 Hz, *J* = 14.3 Hz, 1H, H-5’’), 4.46 (pq, *J* = 3.2 Hz, 1H, H-4’), 4.86 (br s, 1H, NHBoc), 4.89 (dd, *J* = 2.6 Hz, *J* = 6.2 Hz, 1H, H-3’), 5.45 (dd, *J* = 4.2 Hz, *J* = 6.2 Hz, 1H, H-2’), 6.09 (d, *J* = 4.2 Hz, 1H, H-1’), 6.40 (br s, 2H, NH_2_), 7.64 (br s, 1H, NHCO), 8.35 (s, 1H, H-2); ^13^C NMR (100 MHz, CDCl_3_): *δ* 25.41 (CH_3_ isop), 26.04 (CH_2_), 27.46 (CH_3_ isop), 28.38 (3 CH_3_ Boc), 33.49 (***CH_2_***CO), 39.94 (***CH_2_***NHBoc), 40.93 (C-5’), 79.22 (Cq Boc), 81.49 (C-3’), 82.18 (C-2’), 83.95 (C-4’), 91.98 (C-1’), 114.83 (Cq isop), 120.51 (C-5), 127.79 (C-8), 150.17 (C-4), 151.59 (C-2), 153.96 (C-6), 156.23 (CO Boc), 173.01 (CONH); HRMS (ESI-TOF): *m/z* calcd for [C_22_H_32_BrN_7_O_6_+H]^+^ 570.1675, found 570.1683.

*5’-O-[3-(5’-(4-(N-tert-Butyloxycarbonyl-amino)butylamido)-5’-deoxy-2’,3’-O-isopropylideneadenosine-8-yl)prop-2-yn-1-yl]-2’,3’-O-isopropylideneadenosine (**15**).* To a solution of bromide 7 (0.19 g, 0.34 mmol) and alkyne 8c [18] (0.18 g, 0.51 mmol) in THF (4.0 mL) was added NEt_3_ (0.14 mL, 1.02 mmol). The stirred mixture was degassed by vacuum/argon purging (3 times) before adding sequentially CuI (7 mg, 0.036 mmol) and Pd(PPh_3_)_4_ (20 mg, 0.017 mmol), followed by another 15 min argon degassing. After stirring for 3 h at 60 °C, reaction was incomplete (TLC monitoring), Pd(PPh_3_)_4_ (40 mg, 0.034 mmol) was added. After 3 h at 60 °C, volatiles were removed and the residue was purified by flash column chromatography (18 g SiO_2_, 0 to 7% methanol in dichloromethane) to yield 15 (74 mg, 25 %) as a pale orange foam. ^1^H NMR (400 MHz, DMSO-d_6_): δ 1.28 (s, 3H, CH_3_ isop), 1.34 (s, 3H, CH_3_ isop), 1.35 (s, 9H, CH_3_ Boc), 1.48 (s, 3H, CH_3_ isop), 1.55 (s, 3H, CH_3_ isop), 1.58 (quint, J = 7.2 Hz, 2H, CH_2_), 2.04-2.08 (m, 2H, CH_2_CO), 2.89 (q, J = 6.6 Hz, 2H, CH_2_CO), 3.30-3.40 (m, 2H, H-5’A and H-5”A), 3.72 (dd, J = 5.0 Hz, J = 10.3 Hz, 1H, H-5’B), 3.80 (dd, J = 5.7 Hz, J = 10.3 Hz, 1H, H-5’’B), 4.14-4.18 (m, 1H, H-4’A), 4.36-4.40 (m, 1H, H-4’B), 4.58 (s, 2H, CH_2_C≡), 4.95 (dd, J = 3.4 Hz, J = 6.3 Hz, 1H, H-3’A), 5.02 (dd, J = 3.0 Hz, J = 6.2 Hz, 1H, H-3’B), 5.43 (dd, J = 2.6 Hz, J = 6.2 Hz, 1H, H-2’B), 5.51 (dd, J = 2.7 Hz, J = 6.3 Hz, 1H, H-2’A), 6.11 (d, J = 2.7 Hz, 1H, H-1’A), 6.19 (d, J = 2.6 Hz, 1H, H-2’B), 6.73 (br, 1H, NHBoc), 7.54 (br, 2H, NH_2_), 7.65 (br, 2H, NH_2_), 8.03 (t, J = 5.7 Hz, 1H, NHCO), 8.22 (s, 1H, H-2B, 8.24 (s, 1H, H-2A), 8.36 (s, 1H, H-8B); ^13^C NMR (100 MHz, DMSO-d_6_): δ 25.66 (2 CH_3_ isop), 26.12 (CH_2_), 27.50 (2 CH_3_ isop), 28.69 (3 CH_3_ Boc), 33.12 (CH_2_CO), 39.82 (CH_2_NHBoc), 41.06 (C-5’A), 58.67 (C≡C-CH_2_), 70.33 (C-5’B), 75.50 (C≡C-CH_2_), 77.85 (Cq Boc), 81.91 (C-3’B), 82.37 (C-3’A), 82.96 (C-2’A), 83.80 (C-2’B), 84.81 (C-4’B), 85.25 (C-4’A), 89.76 (C-1’A and C-1’B), 92.75 (C≡C-CH_2_), 113.90 (Cq isop), 114.01 (Cq isop), 119.44 (C-5A and C-5B), 132.57 (C-8A), 140.45 (C-8B), 148.78 (C-4A), 149.24 (C-4B), 152.24 (C-2A), 154.24 (C-2B), 155.76 (C-6B), 156.03 (C-6A), 156.38 (CO Boc), 172.60 (CONH); HRMS (ESI-TOF): *m/z* calcd for [C_38_H_50_N_12_O_10_+H]^+^ 835.3851, found 835.3840.

*5’-O-[3-(5’-(4-Aminobutylamido)-5’-deoxy-adenosin-8-yl)prop-2-yn-1-yl]-adenosine **(4).*** Compound **15** (64 mg, 0.08 mmol) was dissolved in an ice-cold solution of 50% aqueous TFA (5.0 mL) and the reaction mixture was allowed to warm to room temperature. After stirring for 3 h at room temperature, water was added and the reaction mixture was lyophilized. Purification of the residue by reverse phase HPLC (5 to 25% acetonitrile in 10 mM TEAA buffer over 15 min) yielded **4** (22.6 mg, 45%) as acetate salt. *t*_R_ = 10.4 min; ^1^H NMR (400 MHz, DMSO-*d_6_*): *δ* 1.68 (quint, *J* = 7.2 Hz, 2H, CH_2_), 1.79 (s, 1.88 H, CH_3_ Ac), 2.20 (t, *J* = 6.2 Hz, 2H, ***CH_2_***CO), 2.65 (t, *J* = 6.2 Hz, 2H, ***CH_2_***NH_2_), 3.38 (dt, *J* = 4.8 Hz, *J* = 14.0 Hz, 1H, H-5’A), 3.47 (dt, *J* = 5.8 Hz, *J* = 14.0 Hz, 1H, H-5”A), 3.77 (dd, *J* = 5.6 Hz, *J* = 10.6 Hz, 1H, H-5’B), 3.85 (dd, *J* = 3.7 Hz, *J* = 10.6 Hz, 1H, H-5”B), 3.98-4.01 (m, 1H, H-4’A), 4.08-4.13 (m, 2H, H-3’A and H-4’B), 4.20 (t, *J* = 4.7 Hz, 1H, H-3’B), 4.61-4.63 (m, 3H, H-2’B and CH_2_C≡), 5.01-5.04 (m, 1H, H-2’A), 5.92 (d, *J* = 5.3 Hz, 1H, H-1’B), 5.94 (d, *J* = 6.4 Hz, 1H, H-1’A), 7.25 (s, 2H, NH_2_), 7.62 (s, 2H, NH_2_), 8.17 (s, 1H, H-2B), 8.24 (s, 1H, H-2A), 8.27 (m, 1H, NHCO), 8.32 (s, 1H, H-8B); ^13^C NMR (100 MHz, DMSO-*d_6_*): *δ* 23.08 (CH_3_ Ac), 26.58 (CH_2_), 33.02 (***CH_2_***CO), 40.02 (***CH_2_***NH_2_), 41.27 (C-5’A), 58.74 (C≡C-***CH_2_***), 70.77 (C-5’B), 71.02 (C-3’B), 71.71 (C-3’A and C-2’A), 73.69 (C-2’B), 75.70 (***C***≡C-CH_2_), 83.28 (C-4’B), 84.55 (C-4’A), 87.96 (C-1’B), 89.67 (C-1’A), 92.56 (C≡***C***-CH_2_), 119.50 (C-5B), 119.81 (C-5A), 133.41 (C-8A), 139.84 (C-8B), 149.16 (C-4A), 149.95 (C-4B), 153.18 (C-2B), 154.21 (C-2A), 156.50 (C-6B), 156.59 (C-6A), 172.41 (CONH), 173.60 (CO Ac); HRMS (ESI-TOF): *m/z* calcd for [C_27_H_34_N_12_O_8_+H]^+^ 655.2701, found 655.2708.

*5’-Deoxy-5’-(2-nitrobenzenesulfonamido)-2’,3’-O-isopropylidene-adenosine **(16).*** To a solution of **13** (2.30 g, 7.5 mmol) in pyridine (75 mL) was added 2-nitro-benzenesulfonyl chloride (3.82 g, 17.25 mmol). After stirring for 2 h at room temperature, volatiles were removed and the residue was purified by flash column chromatography (240 g SiO_2_, 3% isocratic methanol in dichloromethane) to yield compound **16** (3.25 g, 88%) as a white solid. ^1^H NMR (400 MHz, DMSO-*d*_6_): *δ* 1.29 (s, 3H, CH_3_ isop), 1.52 (s, 3H, CH_3_ isop), 3.19-3.33 (m, 2H, H-5’ and H-5’’), 4.24 (td, *J* = 2.9 Hz, *J* = 5.7 Hz, 1H, H-4’), 4.95 (dd, *J* = 2.9 Hz, *J* = 6.2 Hz, 1H, H-3’), 5.37 (dd, *J* = 2.9 Hz, *J* = 6.2 Hz, H-2’), 6.12 (d, *J* = 2.9 Hz, H-1’), 7.38 (s, 2H, NH_2_), 7.70-7.74 (m, 1H, H-4 Ns), 7.78-7.82 (m, 1H, H-3 Ns), 7.85-7.87 (m, 1H, H-5 Ns), 7.91-7-93 (m, 1H, H-2 Ns), 8.13 (s, 1H, H-8), 8.29 (s, 1H, H-2), 8.60 (br s, 1H, NH-5’); ^13^C NMR (100 MHz, DMSO-*d*_6_): *δ* 25.60 (CH_3_ isop), 27.42 (CH_3_ isop), 45.12 (C-5’), 82.01 (C-3’), 83.33 (C-2’), 84.68 (C-4’), 90.00 (C-1’), 133.94 (Cq isop), 299.82 (C-5), 124.83 (C-3 Ns), 129.90 (C-6 Ns), 132.93 (C-5 Ns), 133.01 (C-1 Ns), 134.50 (C-4 Ns), 140.63 (C-8), 148.00 (C-2 Ns), 148.90 (C-4), 153.05 (C-2), 156.71 (C-6); HRMS (ESI-TOF): *m/z* calcd for [C_19_H_21_N_7_O_7_S+H]^+^ 492.1302 found, 492.1302.

*5’-N-(N-tert-Butyloxycarbonyl-aminopropyl)amino-5’-deoxy-2’,3’-O-isopropylidene-adenosine **(17)***. To a solution of **17** (0.80 g, 1.63 mmol) in DMF was added K_2_CO_3_ (0.68 g, 4.88 mmol) followed by *tert*-butyl-3-bromopropylcarbamate (prepared according reported procedures) [25] (0.47 g, 1.95 mmol). After stirring overnight at 50 °C, alkylation was incomplete and *tert*-butyl-3-bromopropylcarbamate (0.23 g, 0.98 mmol) was further added. After heating at 50 °C for 24 h, thiophenol (0.36 mL, 3.26 mmol) was added and stirring was maintained for 18 h at room temperature. Volatiles were then removed under reduced pressure and the residue purified by flash column chromatography (85 g SiO_2_ 70–230 mesh, 5 to 11% methanol in dichloromethane to yield compound **17** (0.61 g, 79%) as a white foam. ^1^H NMR (400 MHz, DMSO-*d*_6_): *δ* 1.33 (s, 3H, CH_3_ isop), 1.36 (s, 9H, CH_3_ Boc), 1.48 (quint, *J* = 6.8 Hz, 2H, CH_2_), 1.54 (s, 3H, CH_3_ isop), 2.46-2.51 (m, 2H, ***CH_2_***NH), 2.66 (dd, *J* = 6.1 Hz, *J* = 12.2 Hz, 2H, H-5’), 2.73 (dd, *J* = 5.9 Hz, *J* = 12.2 Hz, 2H, H-5”), 2.93 (br q, *J* = 6.8 Hz, 2H, ***CH_2_***NHBoc), 4.21 (td, *J* = 2.8 Hz, *J* = 5.9 Hz, 1H, H-4’), 4.97 (dd, *J* = 6.3 Hz, *J* = 2.8 Hz, 1H, H-3’), 5.45 (dd, *J* = 3.0 Hz, *J* = 6.3 Hz, 1H, H-2’), 6.09 (d, *J* = 3.0 Hz, 1H, H-1’), 6.75 (br, 1H, NHBoc), 7.30 (s, 2H, NH_2_), 8.16 (s, 1H, H-8), 8.35 (s, 1H, H-2); ^13^C NMR (100 MHz, DMSO-*d*_6_): *δ* 25.72 (CH_3_ isop), 27.53 (CH_3_ isop), 28.72 (3 CH_3_ Boc), 30.08 (CH_2_), 38.51 (***CH_2_***NHBoc), 47.26 (***CH_2_***NH), 51.47 (C-5’), 77.81 (Cq Boc), 82.65 (C-3’), 83.20 (C-2’), 85.33 (C-4’), 89.75 (C-1’), 113.68 (Cq isop), 119.71 (C-5), 140.42 (C-8), 149.41 (C-4), 153.15 (C-2), 156.07 (CO Boc), 156.62 (C-6); HRMS (ESI-TOF): *m/z* calcd for [C_21_H_33_N_7_O_5_+H]^+^ 464.2621, found 464.2628.

*5’-N-(N-tert-Butyloxycarbonyl-aminopropyl)propargylamino-5’-deoxy-2’,3’-O-isopropylidene-adenosine **(18).*** To a solution of **17** (0.60 g, 1.3 mmol) in DMF (13 mL), were added DIEA (1.35 mL, 7.7 mmol) and propargyl bromide (80% in toluene, 0.79 mL, 7.10 mmol). After stirring for 2 h at room temperature, the reaction was diluted with ethyl acetate (100 mL) and washed with water (2 x 50 mL). Organic layer was dried over Na_2_SO_4_, filtered and concentrated under reduced pressure. The residue was purified by flash column chromatography (20 g SiO_2_, 3 to 4% methanol in dichloromethane) to yield compound **18** (0.53 g, 82%) as a white foam. ^1^H NMR (400 MHz, DMSO-*d*_6_): *δ* 1.33 (s, 3H, CH_3_ isop), 1.37 (s, 9H, CH_3_ Boc), 1.46 (quint, *J* = 6.8 Hz, 2H, CH_2_), 1.54 (s, 3H, CH_3_ isop), 2.40 (t, *J* = 6.8 Hz, 2H, ***CH_2_***N), 2.51-2.55 (m, 1H, H-5’), 2.67 (dd, *J* = 7.6 Hz, *J* = 13.1 Hz, 1H, H-5”), 2.84-2.99 (m, 2H, ***CH_2_***NHBoc), 3.01 (t, *J* = 2.2 Hz, 1H, HC≡C), 3.29-3.41 (m, 2H, ***CH_2_***C≡), 4.22 (td, *J* = 2.8 Hz, *J* = 6.9 Hz, 1H, H-4’), 4.99 (dd, *J* = 2.8 Hz, *J* = 6.3 Hz, 1H, H-3’), 5.49 (dd, *J* = 2.4 Hz, *J* = 6.3 Hz, 1H, H-2’), 6.14 (dd, *J* = 2.4 Hz, 1H, H-1’), 6.72 (br, 1H, NHBoc), 7.30 (s, 2H, NH_2_), 8.18 (s, 1H, H-8), 8.31 (s, 1H, H-2); ^13^C NMR (100 MHz, DMSO-*d*_6_): *δ* 25.67 (CH_3_ isop), 27.43 (CH_3_ isop), 27.79 (CH_2_), 28.73 (3 CH_3_ Boc), 38.39 (***CH_2_***NHBoc), 42.40 (C≡C-***CH_2_***), 51.53 (***CH_2_***N), 55.32 (C-5’), 76.01 (***HC***≡C-CH_2_), 77.84 (Cq Boc), 79.21 (C≡***C***-CH_2_), 83.34 (C-3’), 83.43 (C-2’), 84.75 (C-4’), 89.67 (C-1’), 113.64 (Cq isop), 119.69 (C-5), 140.47 (C-8), 149.28 (C-4), 153.18 (C-2), 156.04 (CO Boc), 156.60 (C-6); HRMS (ESI-TOF): *m/z* calcd for [C_24_H_35_N_7_O_5_+H]^+^ 502.2778, found 502.2770.

*5’-Deoxy-5’-N-(N-tert-Butyloxycarbonyl-aminopropyl)-3-(2’,3’,5’-tri-O-acetyl-adenosine-8-yl)propargyl amino-2’,3’-O-isopropylidene-adenosine **(19**).* To a solution of bromide **6** (0.26 g, 0.55 mmol) and alkyne **18** (0.41 g, 0.82 mmol) in THF (16.5 mL) was added NEt_3_ (0.23 mL, 1.65 mmol). The stirred mixture was degassed with argon (3 times) before adding sequentially CuI (10 mg, 0.05 mmol) and Pd(PPh_3_)_4_ (32 mg, 0.028 mmol), followed by argon degassing. After stirring for 4 h at 60 °C, a second addition of Pd(PPh_3_)_4_ (32 mg, 0.028 mmol) was made. After 18 h at 60 °C, crude was filtered over Celite and volatiles were removed under reduced pressure. The residue was purified by flash column chromatography (31 g SiO_2_, 0 to 7% methanol in dichloromethane) to yield 7 (0.31 g, 63%) as a white foam. ^1^H NMR (400 MHz, DMSO-d_6_): δ 1.34 (s, 3H, CH_3_ isop), 1.37 (s, 9H, CH_3_ Boc), 1.55 (s, 3H, CH_3_ isop), 1.51-1.60 (m, 2H, CH_2_), 1.95 (s, 3H, COCH_3_), 1.96 (s, 3H, COCH_3_), 2.06 (s, 3H, COCH_3_), 2.51-2.56 (m, 2H, CH_2_N), 2.66 (dd, J = 6.0 Hz, J = 13.2 Hz, 1H, H-5’B), 2.82 (dd, J = 8.0 Hz, J = 13.2 Hz, 1H, H-5”B), 2.89-3.05 (m, 2H, CH_2_NHBoc), 3.77-3.88 (m, 2H, CH_2_C≡), 4.16 (dd, J = 5.6 Hz, J = 12.0 Hz, 1H, H-5’A), 4.28-4.35 (m, 2H, H-4’A and H-4’B), 4.41 (dd, J = 3.7 Hz, J = 12.0 Hz, 1H, H-5”A), 5.04 (dd, J = 2.8 Hz, J = 6.3 Hz, 1H, H-3’B), 5.51 (dd, J = 2.5 Hz, J = 6.4 Hz, 1H, H-2’B), 5.72 (t, J = 6.0 Hz, 1H, H-3’A), 6.11 (d, J = 4.6 Hz, 1H, H-1’A), 6.17 (d, J = 2.5 Hz, 1H, H-1’B), 6.19 (dd, J = 4.6 Hz, J = 6.0 Hz, 1H, H-2’A), 6.77 (br t, J = 5.4 Hz, 1H, NHBoc), 7.31 (br, 1H, NH_2_), 7.57 (s, 2H, NH_2_), 8.18 (s, 1H, H-2A), 8.19 (s, 1H, H-2B), 8.33 (s, 1H, H-8B); ^13^C NMR (100 MHz, DMSO-d_6_): δ 20.48 (CH_3_), 20.69 (CH_3_), 20.84 (CH_3_), 25.66 (CH_3_ isop), 27.43 (CH_3_ isop), 27.90 (CH_2_), 28.70 (3 CH_3_ Boc), 38.31 (CH_2_NHBoc), 43.16 (C≡C-CH_2_), 51.78 (CH_2_N), 55.35 (C-5’B), 62.86 (C-5’A), 70.24 (C-3’A), 71.71 (C-2’A), 74.34 (C≡C-CH_2_), 77.90 (Cq Boc), 79.69 (C-4’A), 83.26 (C-3’B), 83.43 (C-2’B), 84.33 (C-4’B), 87.30 (C-1′A), 89.71 (C-1′B), 93.23 (C≡C-CH_2_), 113.77 (Cq isop), 119.26 (C-5A), 119.71 (C-5B), 133.28 (C-8A), 140.54 (C-8B), 149.04 (C-4B), 149.24 (C-4A), 153.19 (C-2A), 154.32 (C-2B), 156.09 (CO Boc), 156.43 (C-5B), 156.62 (C-6A), 169.73 (CO), 169.81 (CO), 170.41 (CO); HRMS (ESI-TOF): *m/z* calcd for [C_40_H_52_N_12_O_12_+H]^+^ 893.3906, found 893.3939.

*5’-[N-3-Aminopropyl-N-(3-(adenosin-8-yl)propargyl)amino]-5’-deoxyadenosine **(5).*** To a solution of **7** (0.29 g, 0.33 mmol) in methanol (3.3 mL) was added 28% NH_4_OH (0.80 mL, 13.3 mmol). After stirring for 18 h at room temperature, volatiles were removed under reduced pressure and the crude material was brought to 0 °C before adding an ice-cold solution of TFA (50% in water, 10 mL). After 4 h at room temperature, water was added and the reaction mixture was lyophilized. Purification by reverse phase HPLC (0 to 30% acetonitrile in 10 mM TEAA buffer over 15 min) afforded the fully deprotected compound **5** as acetate salt (0.12 g, 61%) as white powder. *t*_R_ = 10.8 min; ^1^H NMR (400 MHz, DMSO-*d*_6_): *δ* 1.65 (quint, *J* = 6.8 Hz, 2H, CH_2_), 1.78 (s, 3H, CH_3_ Ac), 2.65 (t, *J* = 6.8 Hz, 2H, ***CH_2_***N), 2.70 (t, *J* = 6.8 Hz, 2H, ***CH_2_***NH_2_), 2.80 (dd, *J* = 4.8 Hz, *J* = 13.7 Hz, 1H, H-5’B), 2.90 (dd, *J* = 6.8 Hz, *J* = 13.7 Hz, 1H, H-5”B), 3.53 (dd, *J* = 4.0 Hz, *J* = 12.2 Hz, 1H, H-5’A), 3.69 (dd, *J* = 3.6 Hz, *J* = 12.2 Hz, 1H, H-5”A), 3.80 (s, 2H, CH_2_C≡), 3.96-4.01 (m, 1H, H-4’A), 4.03-4.08 (m, 1H, H-4’B), 4.14 (pt, *J* = 5.0 Hz, 1H, H-3’B), 4.20 (dd, *J* = 2.3 Hz, *J* = 5.1 Hz, 1H, H-3’A), 4.67 (pt, *J* = 5.1 Hz, 1H, H-2’B), 4.98 (dd, *J* = 5.3 Hz, *J*= 6.0 Hz, 1H, H-2’A), 5.89 (d, *J* = 5.4 Hz, 1H, H-1’B), 6.00 (dd, *J* = 6.8 Hz, H-1’A), 7.25 (br, 1H, NH_2_ B), 7.61 (s, 2H, NH_2_ A), 8.15 (s, 1H, H-2A), 8.17 (s, 1H, H-2B), 8.36 (s, 1H, H-8B); ^13^C NMR (100 MHz, DMSO-*d*_6_): *δ* 23.23 (CH_3_ Ac), 28.50 (***CH_2_***N), 38.89 (***CH_2_***NH_2_), 44.80 (C≡C-***CH_2_***), 51.78 (***CH_2_***N), 56.23 (C-5’B), 62.69 (C-5’A), 71.49 (C-3’A), 72.21 (C-3’B and C-2’A), 73.14 (C-2’B), 74.81 (***C***≡C-CH_2_), 83.03 (C-4’B), 87.11 (C-4’A), 88.10 (C-1′B), 90.01 (C-1′A), 92.96 (C≡***C***-CH_2_), 119.64 and 119.67 (C-5A and C-5B), 133.94 (C-8A), 140.24 (C-8B), 148.84 (C-4A), 149.90 (C-4B), 153.14 (C-2B), 153.67 (C-2A), 156.52 (C-6B), 156.54 (C-6A), 173.90 (CO Ac); HRMS (ESI-TOF): *m/z* calcd for [C_26_H_34_N_12_O_7_+H]^+^ 627.2751 found, 627.2731.

### 4.3. K_i_ Measurements

Activity was measured at 30 °C in 50 mM Bis-Tris pH 7.0, 2 mM sodium citrate, 1 mM MgCl_2_. Reaction mixtures contained 0–2 mM NAD, 4 mM MgATP, 5 mM Glucose 6-phosphate, 1 U/mL Glucose 6-phosphate dehydrogenase and 0.1 µM *Lm*NADK1. Steady state rate constants (k_ss_) were calculated by fitting the initial (first 500–1000 s) linear part of the NADP production time courses per NADK. Inhibitors were tested at 4 concentrations: 0, 10, 25 or 50 µM and Ki values were determined using the competitive inhibition equation, k_ss_ = k_cat_ × NAD/(K_M_ × (1 + [inhibitor]/K_i_) + NAD), with GraFit software (v 7.0.3).

## Supplementary Materials

Supplementary File: Copies of ^1^H and ^13^C NMR spectra of prepared compounds.
