# Supplementary material for: New Chemical Probe Targeting Bacterial NAD Kinase
Source: Molecules. 2020 Oct 22;25(21):4893. doi: 10.3390/molecules25214893 (PMC7660225; doi:10.3390/molecules25214893)
Supplement: Supplementary file 1 [file molecules-25-04893-s001.pdf]

molecules

Supplementary Materials

New Chemical Probe Targeting Bacterial NAD Kinase

David A. Clément [1,2,†], Clarisse Leseigneur [2,3,†], Muriel Gelin [4,†], Dylan Coelho [1], Valérie Huteau [1], Corinne Lionne [4], Gilles Labesse [4,*], Olivier Dussurget [2,3 *] and Sylvie Pochet [1,*]

[1] Institut Pasteur, Unité de Chimie et Biocatalyse, UMR3523 CNRS, Paris 75015, France; david.clement@pasteur.fr (D.A.C.); dylan.coelho@etu.parisdescartes.fr (D.C.); valerie.huteau@pasteur.fr (V.H.)

[2] Faculté des Sciences, Université de Paris, Sorbonne Paris Cité, Paris 75013, France; clarisse.leseigneur@pasteur.fr

[3] Institut Pasteur, Unité de Recherche *Yersinia*, Paris 75015, France; olivier.dussurget@pasteur.fr

[4] Centre de Biochimie Structurale (CBS), CNRS, INSERM, Université de Montpellier, Montpellier 34090, France; gelin@cbs.cnrs.fr (M.G.); lionne@cbs.cnrs.fr (C.L.)

* Correspondence: labesse@cbs.cnrs.fr (G.L.); olivier.dussurget@pasteur.fr (O.D.); sylvie.pochet@pasteur.fr (S.P.)

† These authors contributed equally to this work.

Table of contents

1. Numbering of synthesized molecules 3

2. NMR spectra of final compounds 4

Compound **11a** : ^{1}H and ^{13}C spectra. 4

Compound **11a** : HSQC (red) and HMBC (blue) spectra. 5

Compound **11b** : ^{1}H and ^{13}C spectra 6

Compound **11b** : HSQC (red) and HMBC (blue) spectra 7

Compound **8a** : ^{1}H and ^{13}C spectra 8

Compound **8a** : HSQC (red) and HMBC (blue) spectra 9

Compound **8b** : ^{1}H and ^{13}C spectra 10

Compound **8b** : HSQC (red) and HMBC (blue) spectra 11

Compound **12a** : ^{1}H and ^{13}C spectra 12

Compound **12b** : ^{1}H and ^{13}C spectra 13

Compound **2** : ^{1}H and ^{13}C spectra 14

Compound **3** : ^{1}H and ^{13}C spectra 15

Compound **14** : ^{1}H and ^{13}C spectra 16

Compound **7** : ^{1}H and ^{13}C spectra 17

Compound **15** : ^{1}H and ^{13}C spectra 18

Compound **4** : ^{1}H and ^{13}C spectra 19

Compound **16** : ^{1}H and ^{13}C spectra 20

Compound **17** : ^{1}H and ^{13}C spectra 21

Compound **18** : ^{1}H and ^{13}C spectra 22

Compound **19** : ^{1}H and ^{13}C spectra 23

Compound **5** : ^{1}H and ^{13}C spectra 24

1. Numbering of Synthesized Molecules

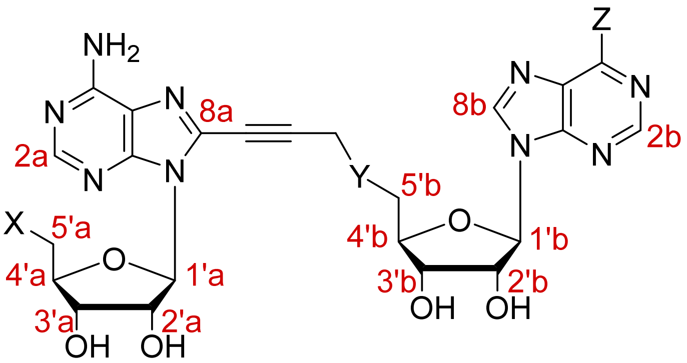

2. NMR spectra of Final Compounds

N[6]-(2-*tert*-Butyloxycarbonyl)aminoethyl-2',3'-*O*-isopropylidene-adenosine **(11a)**.

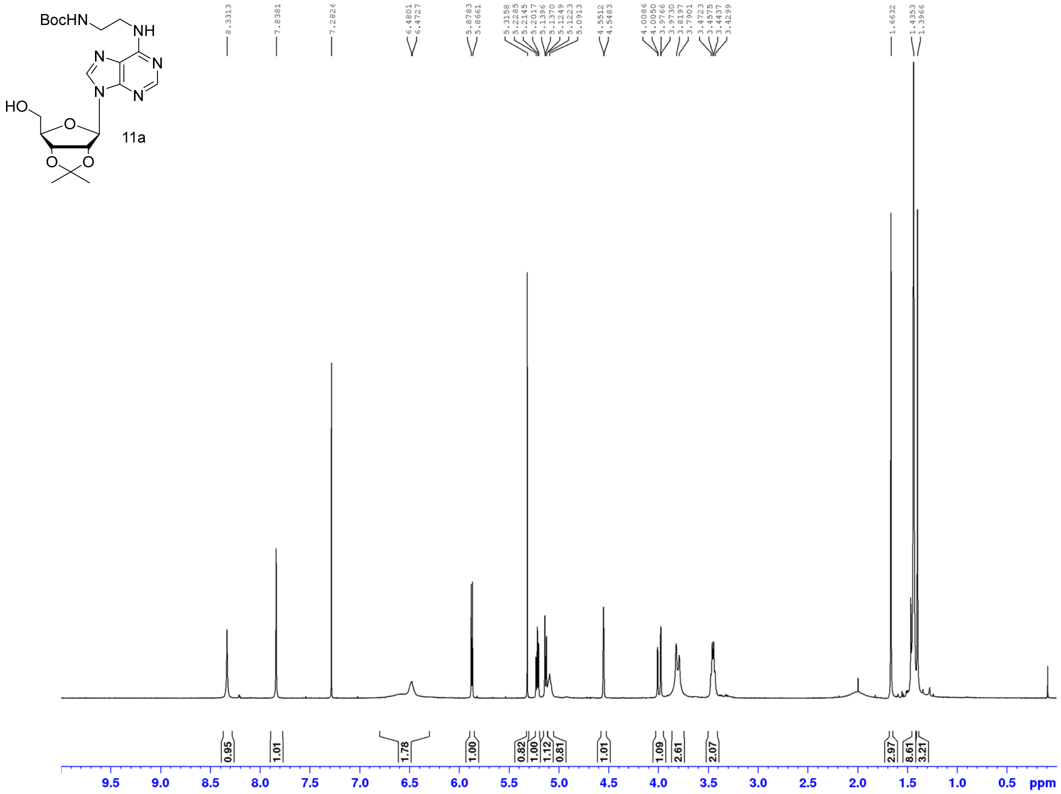

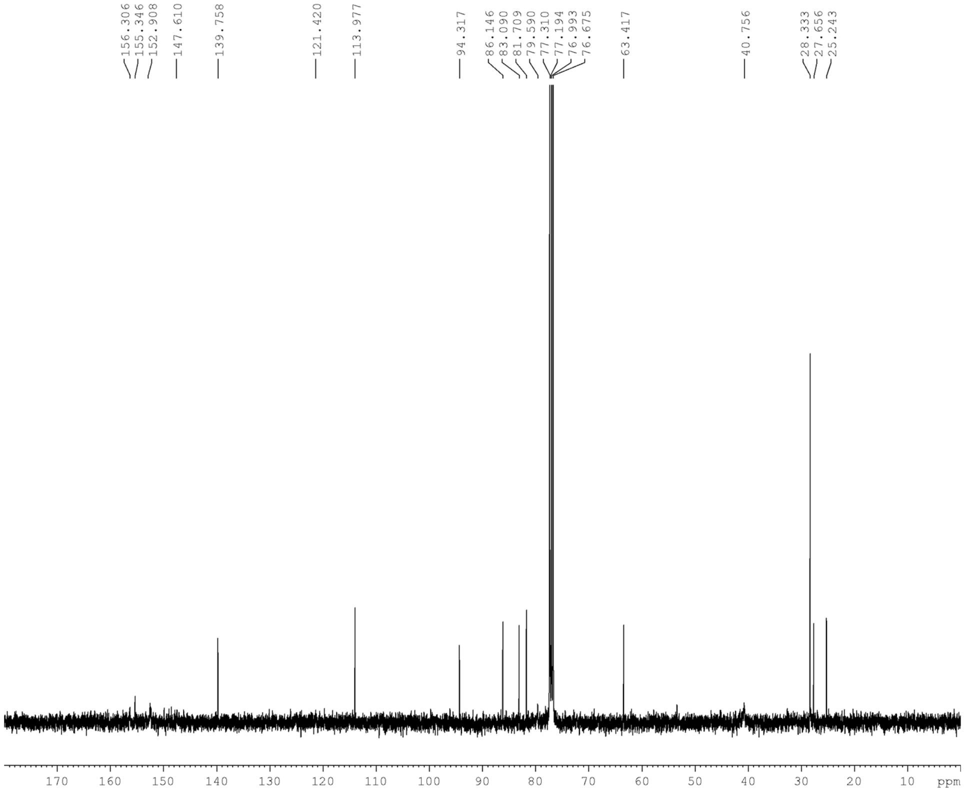

*N*6-(2-*tert*-Butyloxycarbonyl)aminoethyl-2',3'-*O*-isopropylidene-adenosine (**11a**).

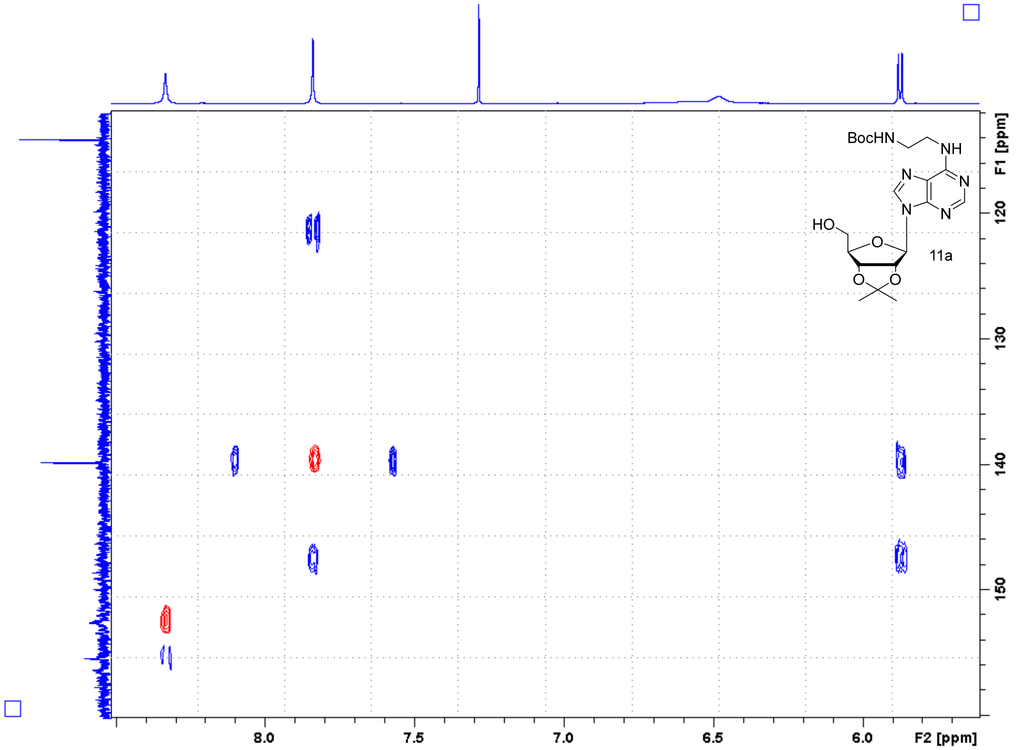

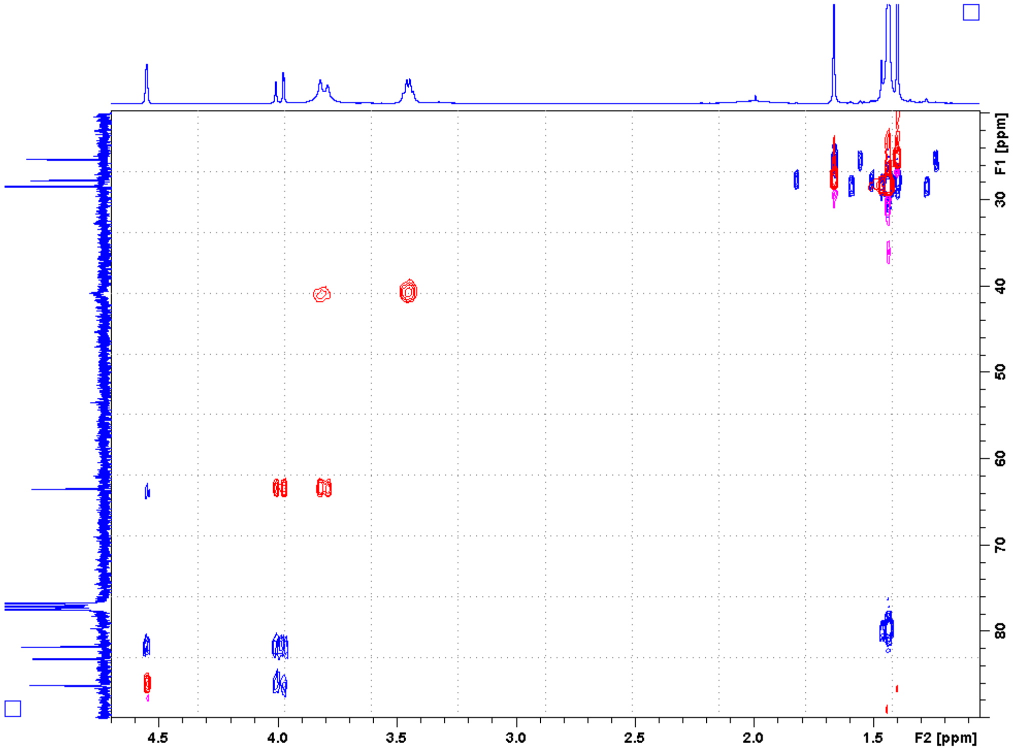

*N*6-(2-*tert*-Butyloxycarbonyl)aminobutyl-2',3'-*O*-isopropylidene-adenosine (**11b**).

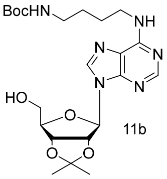

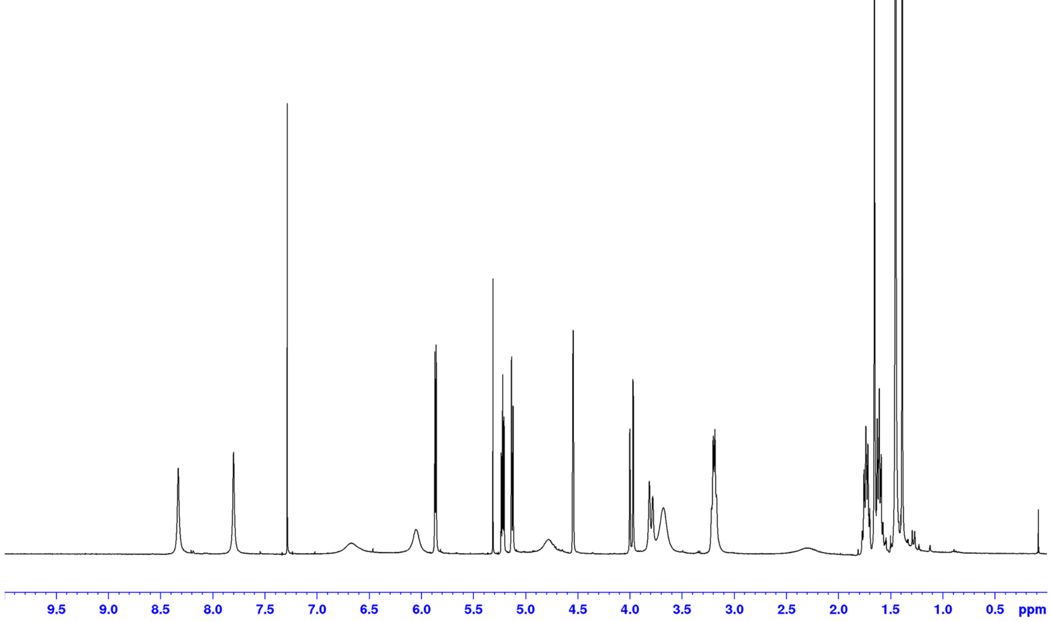

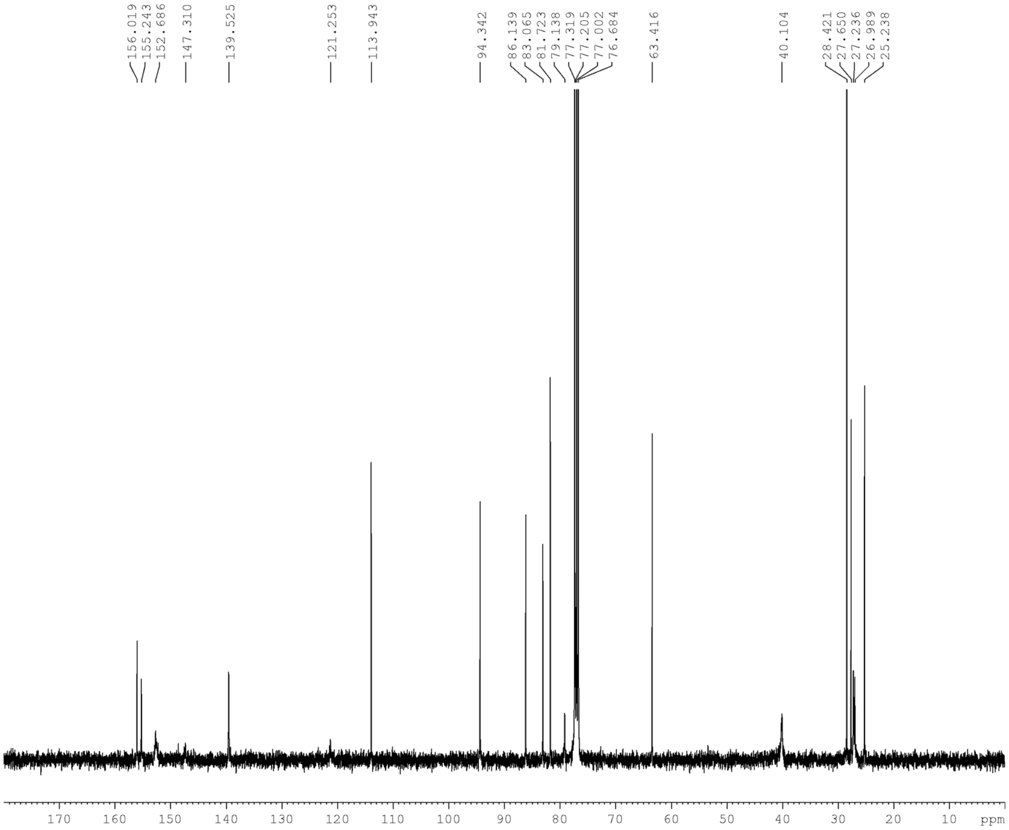

*N*6-(2-*tert*-Butyloxycarbonyl)aminobutyl-2',3'-*O*-isopropylidene-adenosine (**11b**).

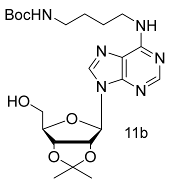

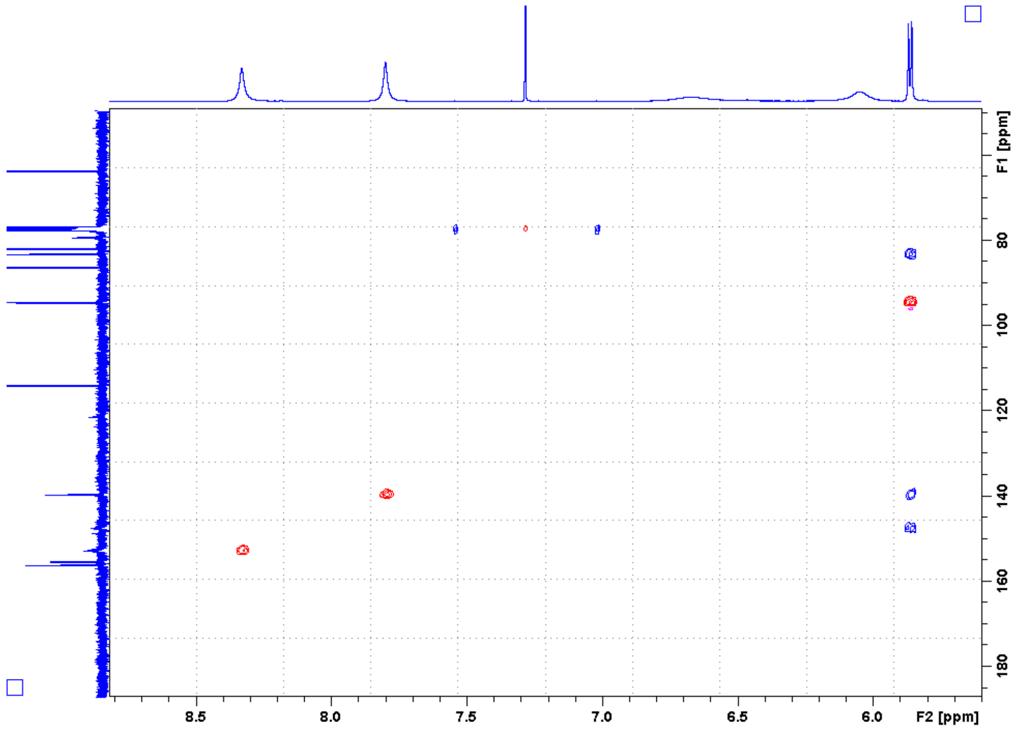

N[6]-(2-*tert*-Butyloxycarbonyl)aminoethyl-2',3'-*O*-isopropylidene-5'-*O*-propargyl-adenosine (**8a**).

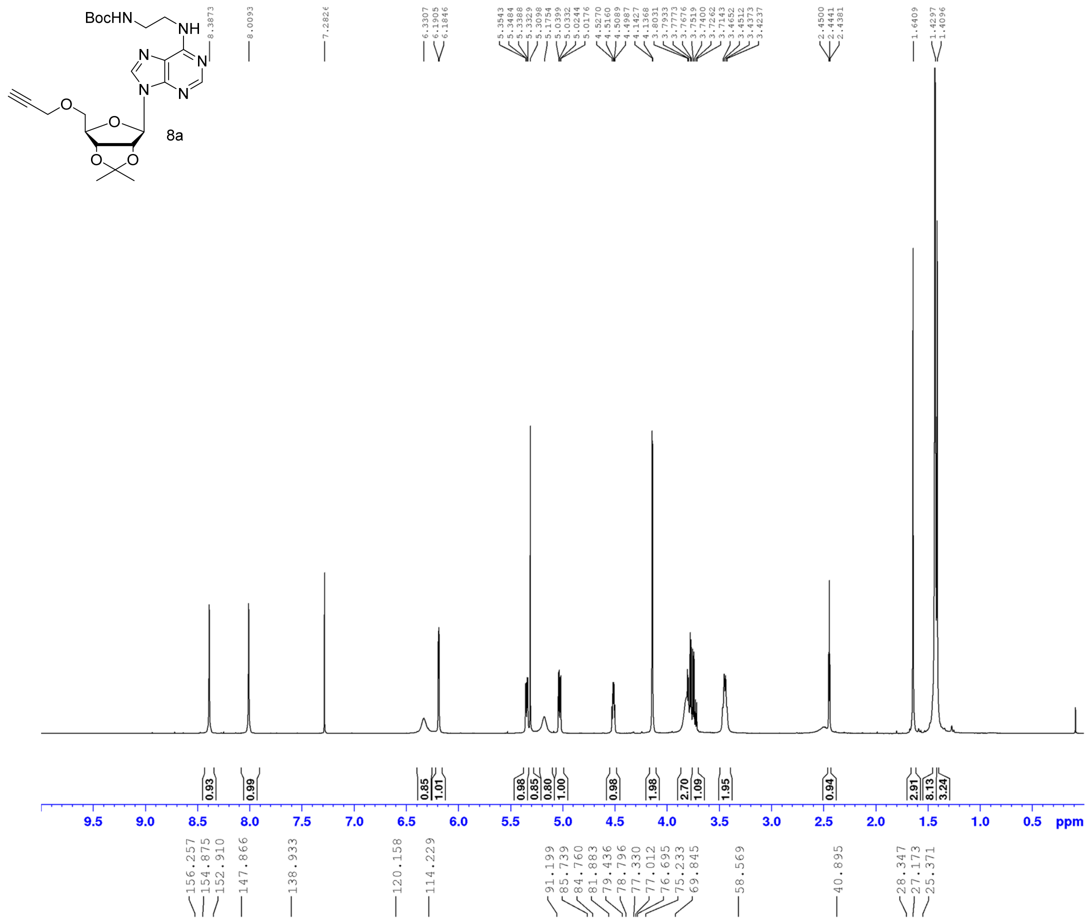

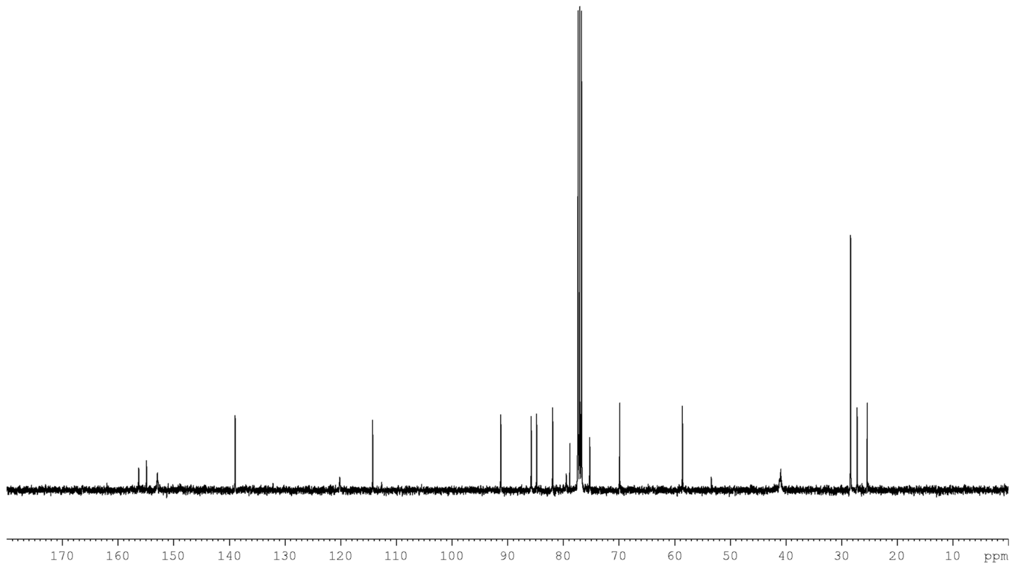

N[6]-(2-*tert*-Butyloxycarbonyl)aminoethyl-2',3'-*O*-isopropylidene-5'-*O*-propargyl-adenosine (**8a**).

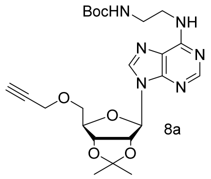

8a

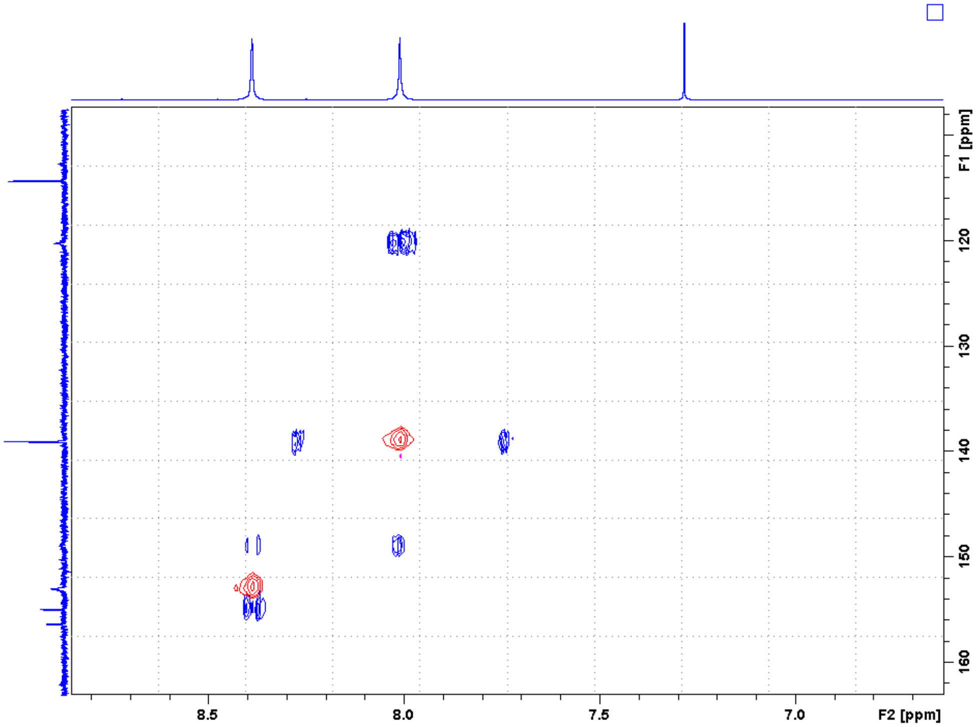

*N*6-(2-*tert*-Butyloxycarbonyl)aminobutyl-2',3'-*O*-isopropylidene-5'-*O*-propargyl-adenosine (**8b**).

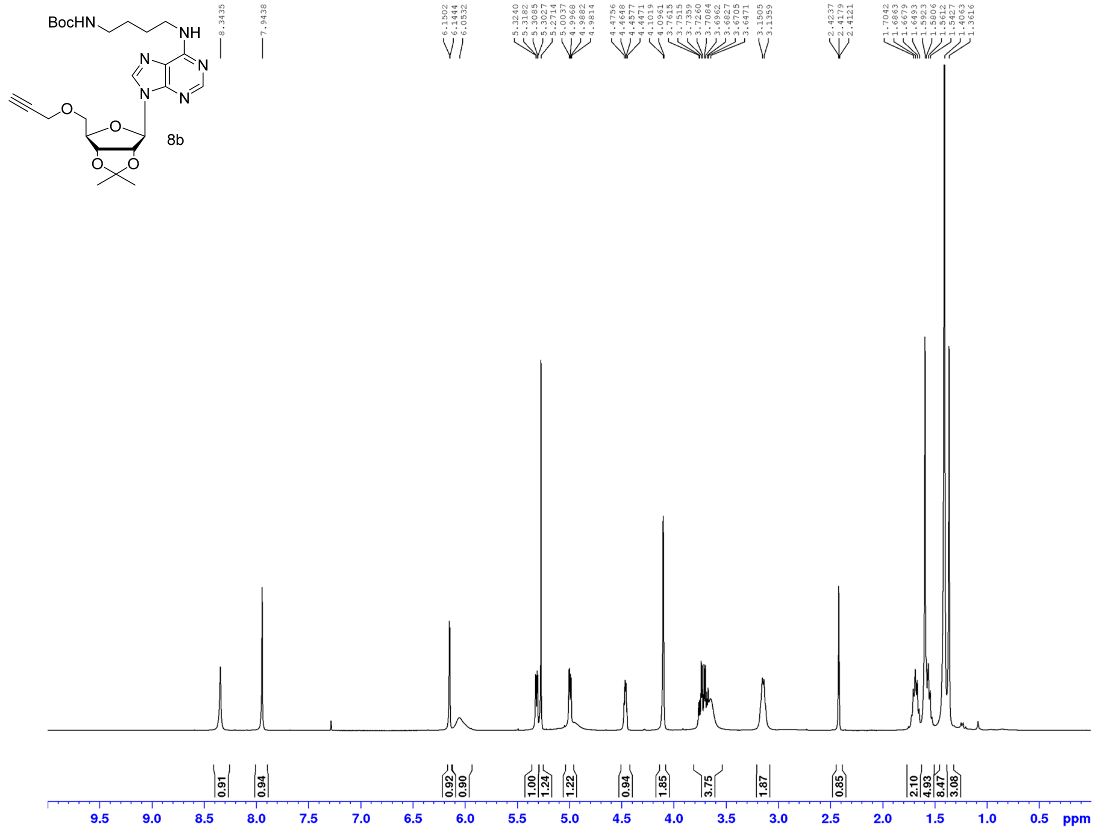

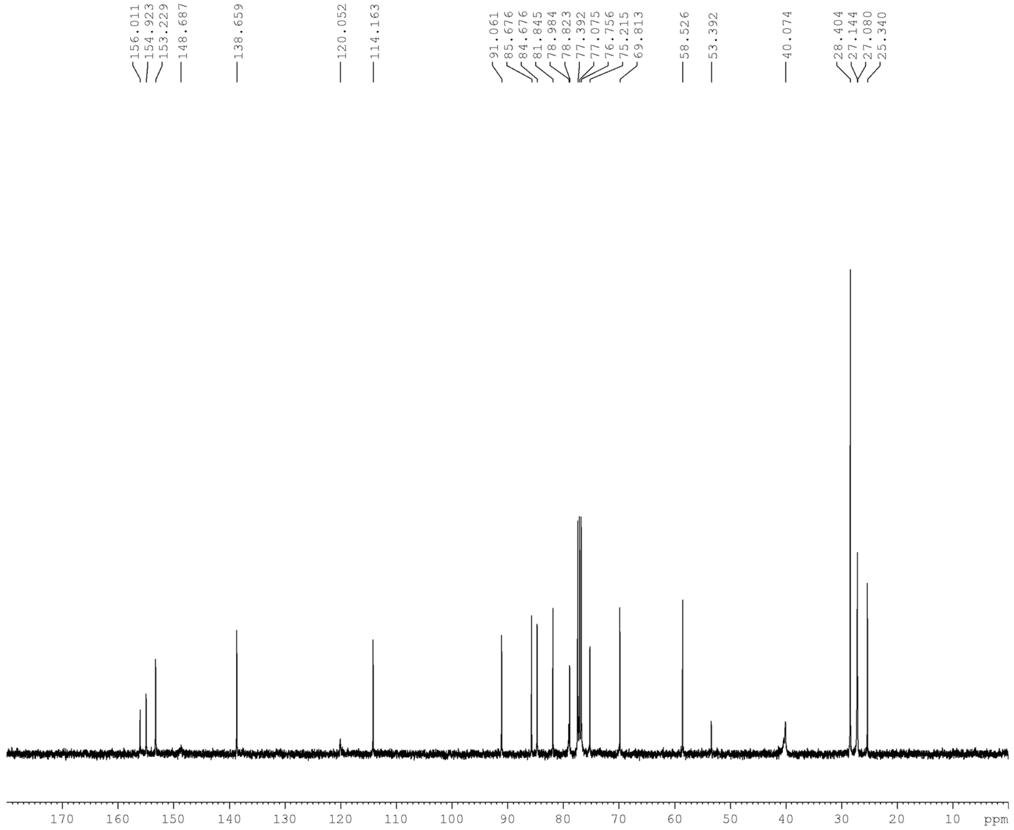

N[6]-(2-*tert*-Butyloxycarbonyl)aminobutyl-2',3'-*O*-isopropylidene-5'-*O*-propargyl-adenosine (**8b**).

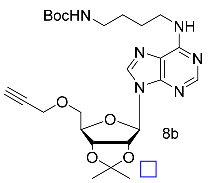

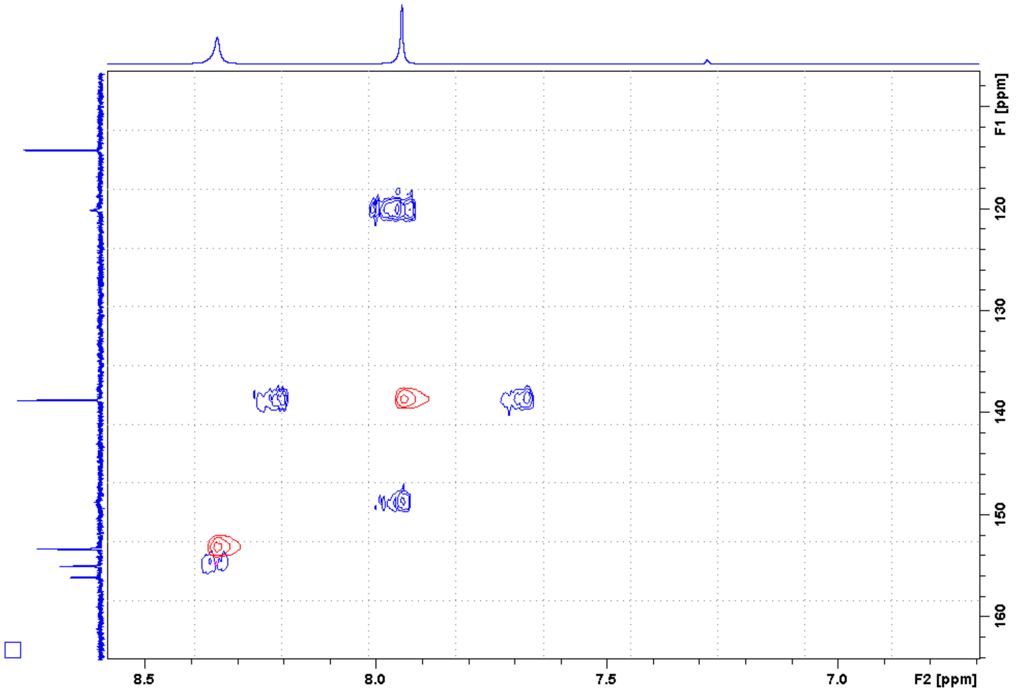

2',3',5'-Tri-*O*-acetyl-8-[3-(*N*[6]-(2-*tert*-butyloxycarbonyl)aminoethyl-2',3'-*O*-isopropylidene-5'-*O*-adenosinyl)propargyl]adenosine (**12a**).

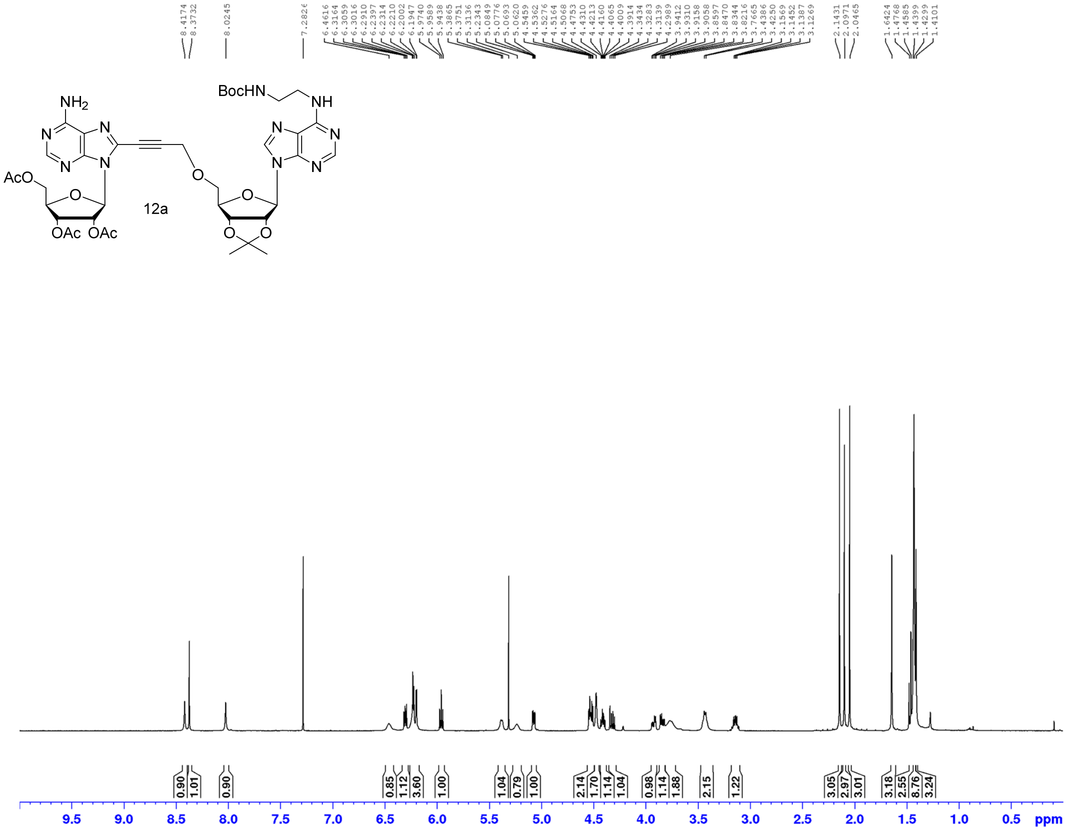

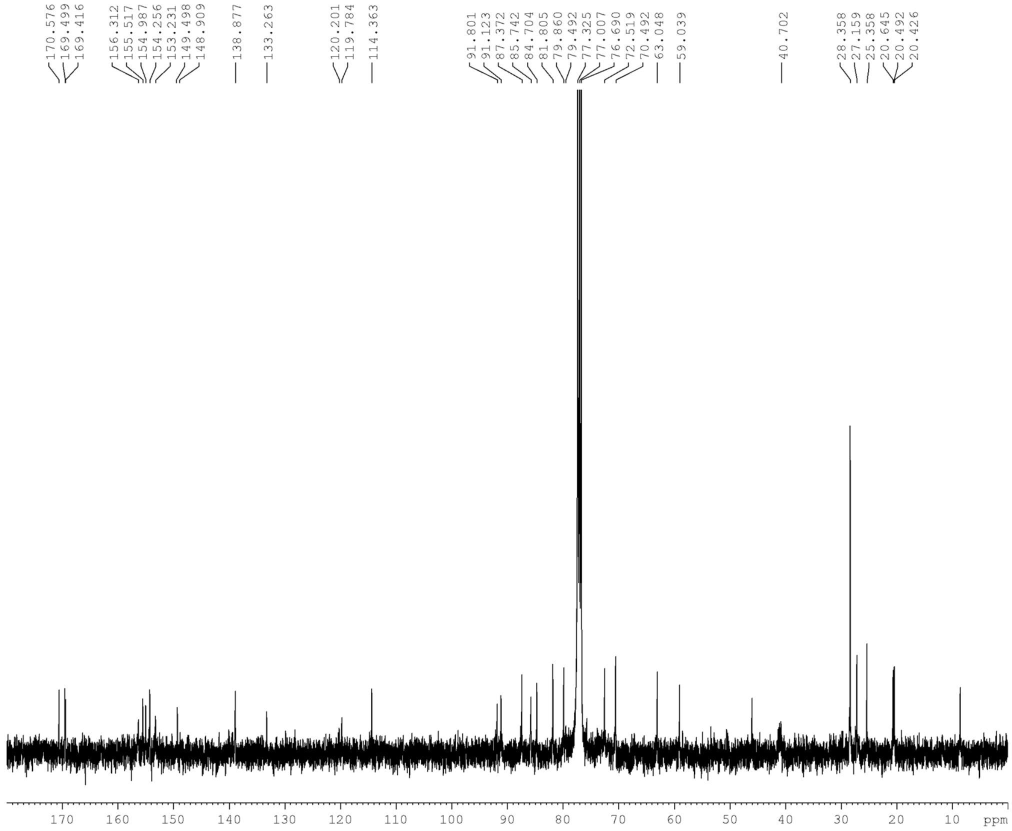

2',3',5'-Tri-*O*-acetyl-8-[3-(*N*[6]-(4-*tert*-butyloxycarbonyl)aminobutyl-2',3'-*O*-isopropylidene-5'-*O*-adenosinyl)propargyl]adenosine (**12b**).

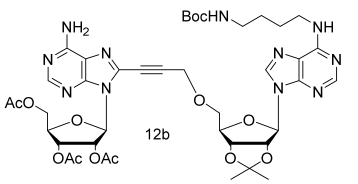

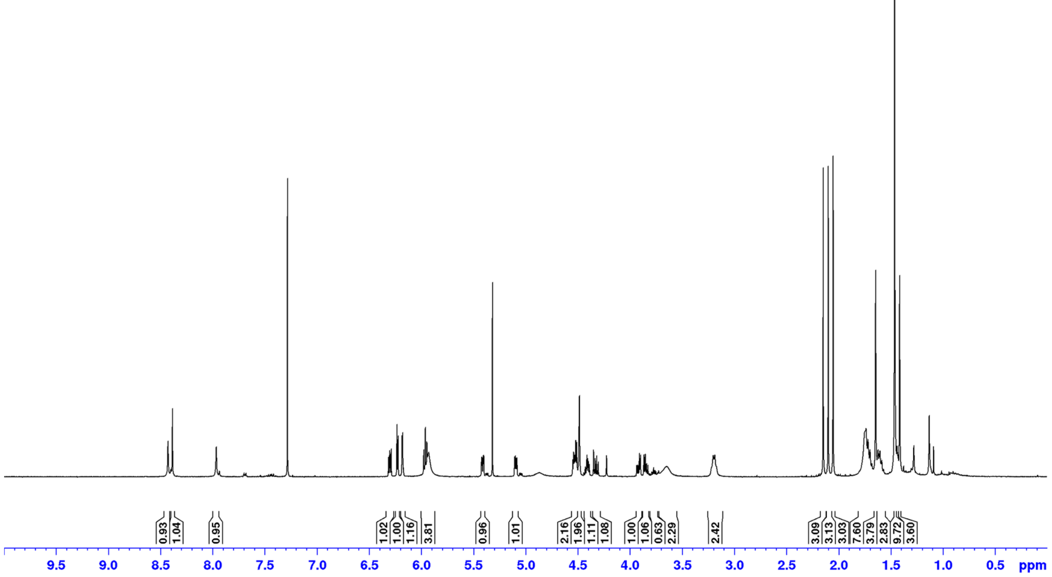

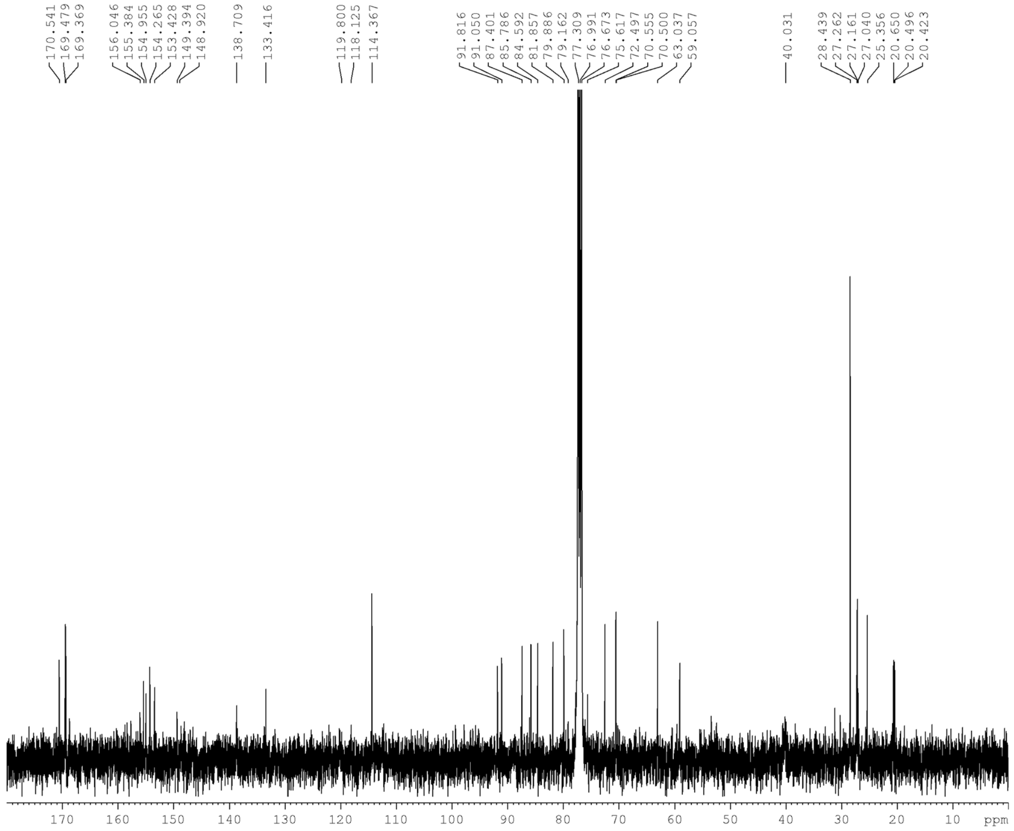

8-[3-(N^6-2-Aminoethyl-5'-*O*-adenosinyl)propargyl]adenosine (**2**).

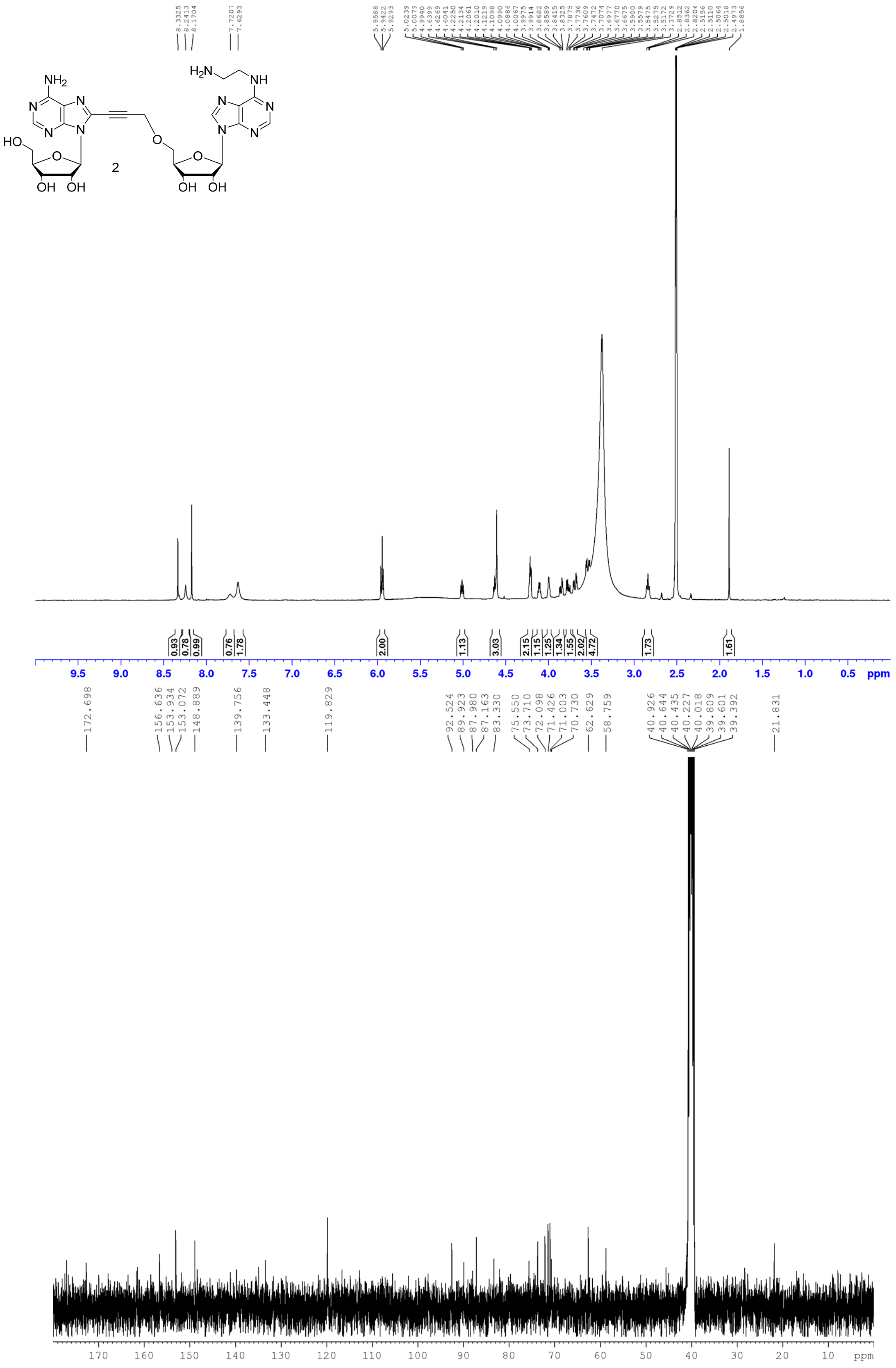

8-[3-(N^6-4-Aminobutyl-5'-*O*-adenosinyl)propargyl]adenosine (**3**).

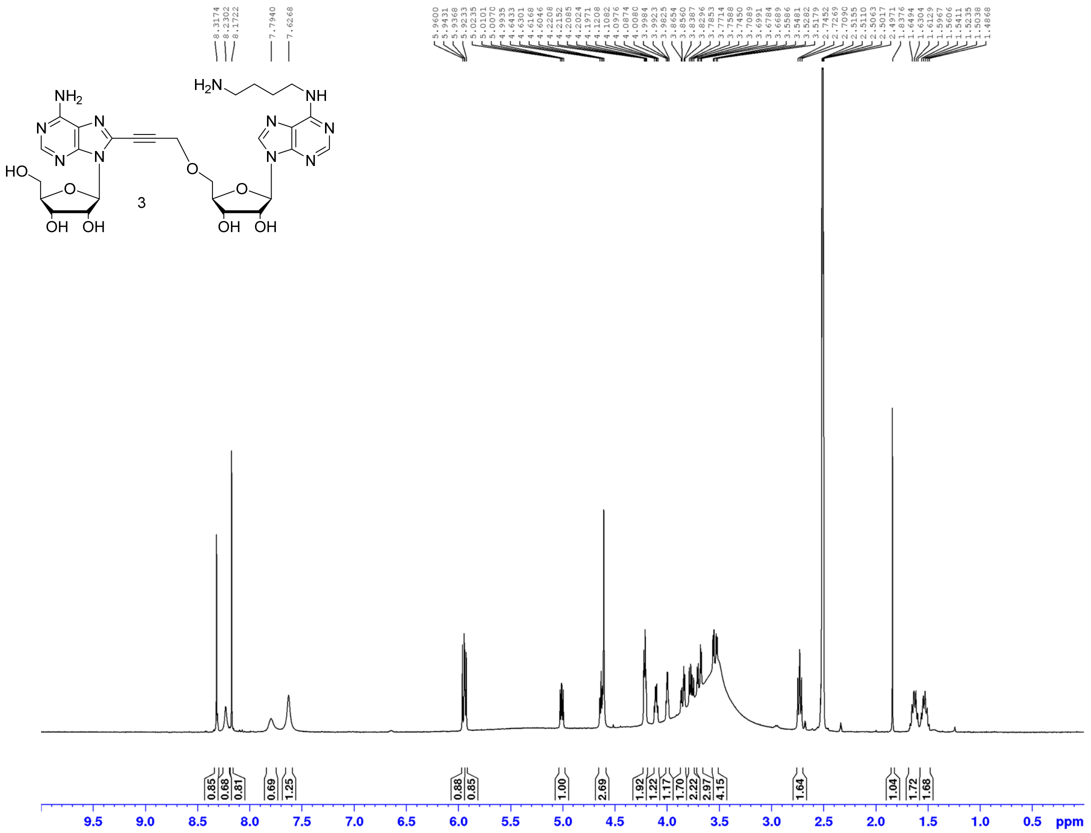

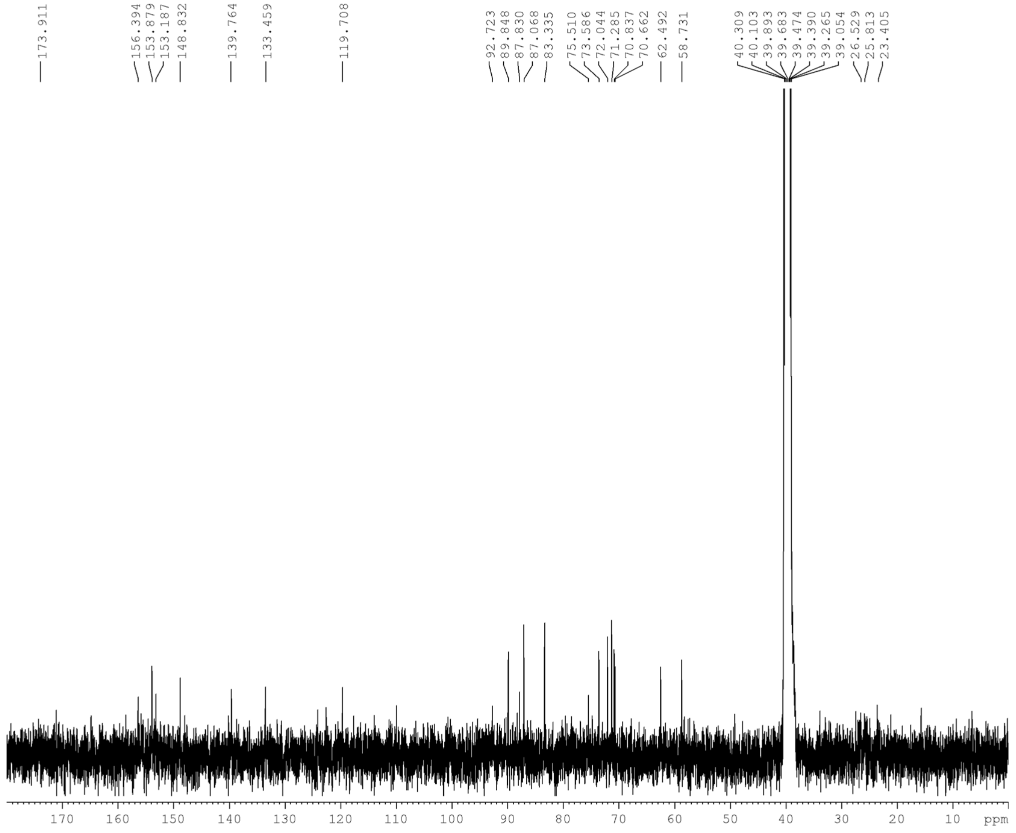

5'-[4-(*N*-*tert*-Butyloxycarbonyl-amino)butylamido]-5'-deoxy-2',3'-*O*-isopropylidene-adenosine (**14**).

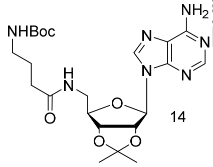

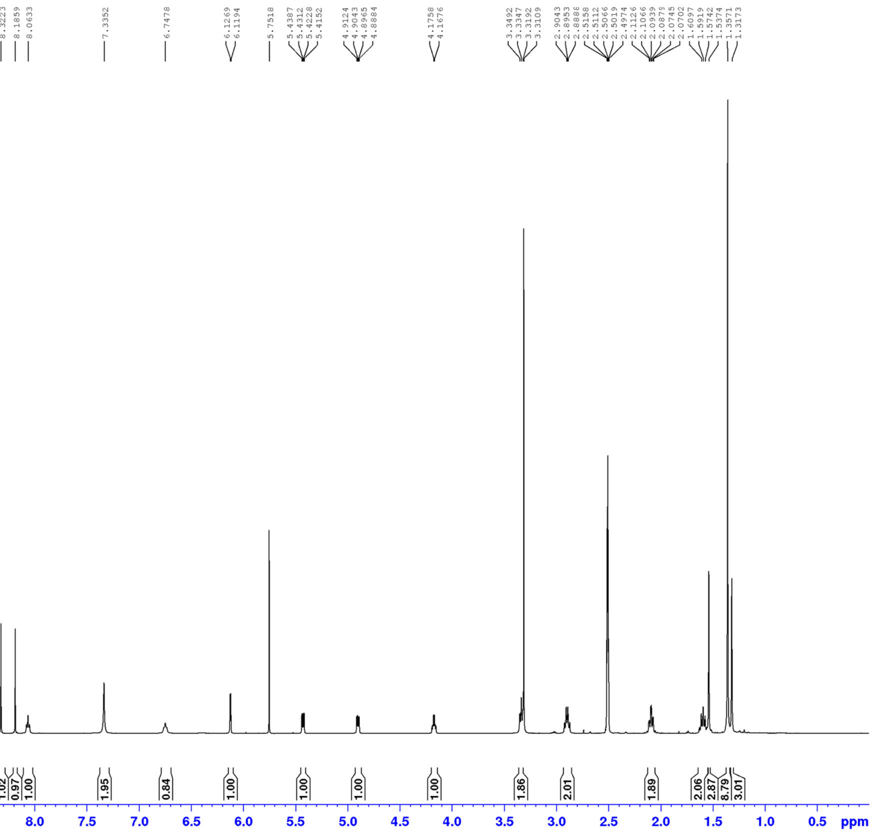

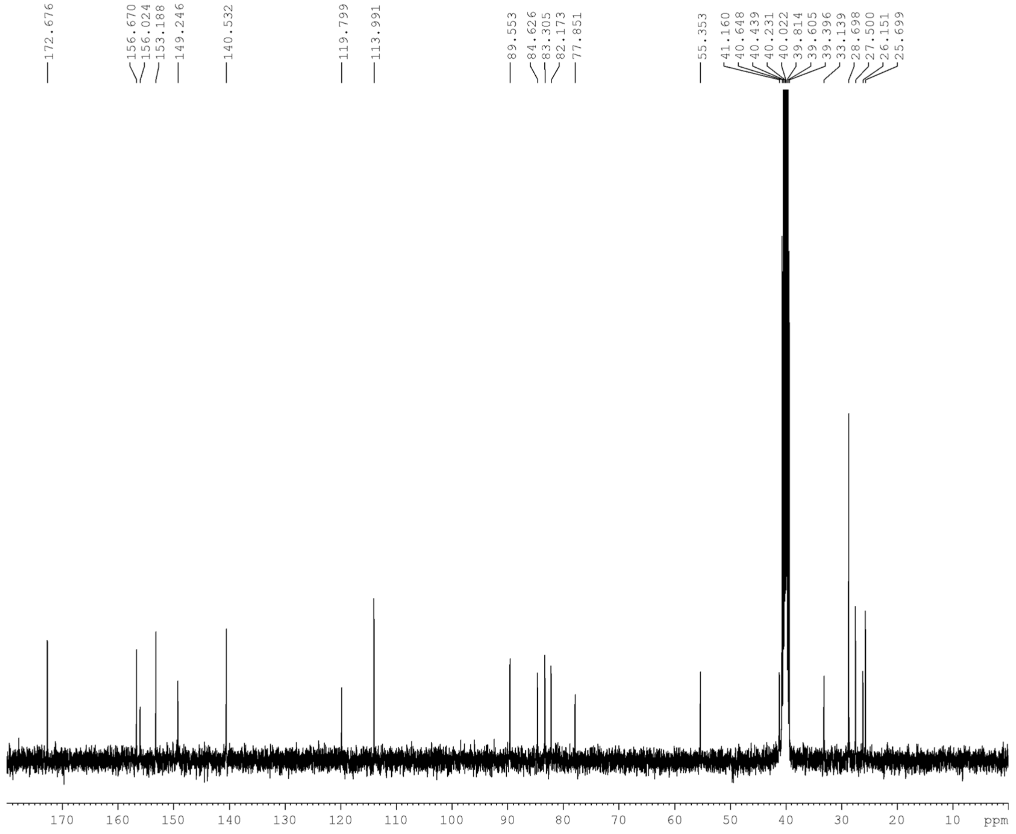

8-Bromo-5'-(4-(*N*-*tert*-butyloxycarbonyl-amino)butylamido)-5'-deoxy-2',3'-*O*-isopropylidene-adenosine (**7**).

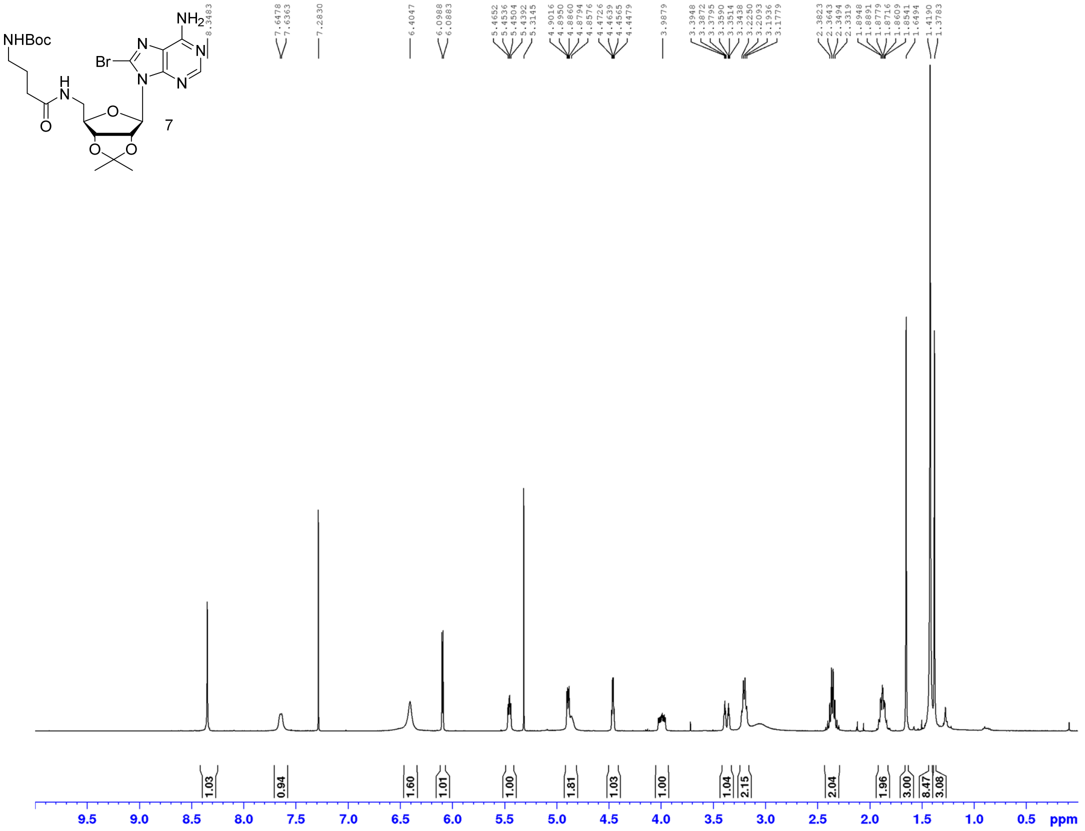

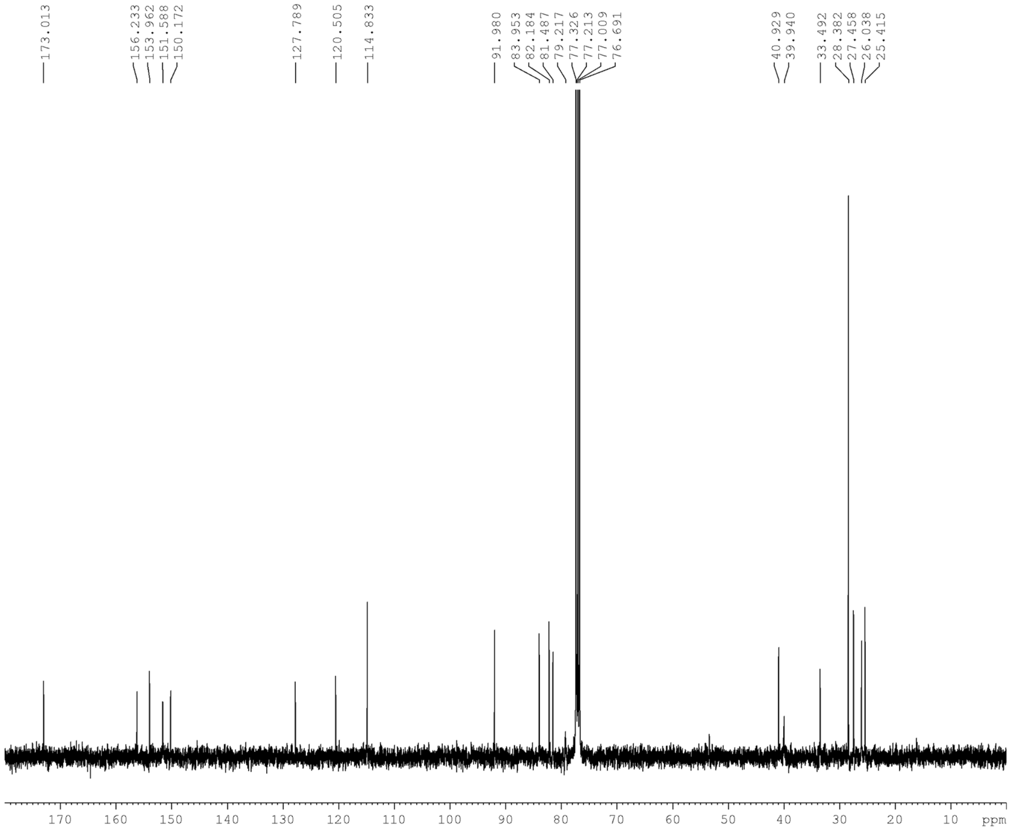

5'-*O*-[3-(5'-(4-(*N*-*tert*-Butyloxycarbonyl-amino)butylamido)-5'-deoxy-2',3'-*O*-isopropylideneadenosine-8-yl)prop-2-yn-1-yl]-2',3'-*O*-isopropylideneadenosine (**15**).

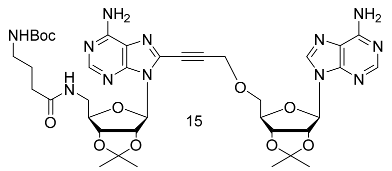

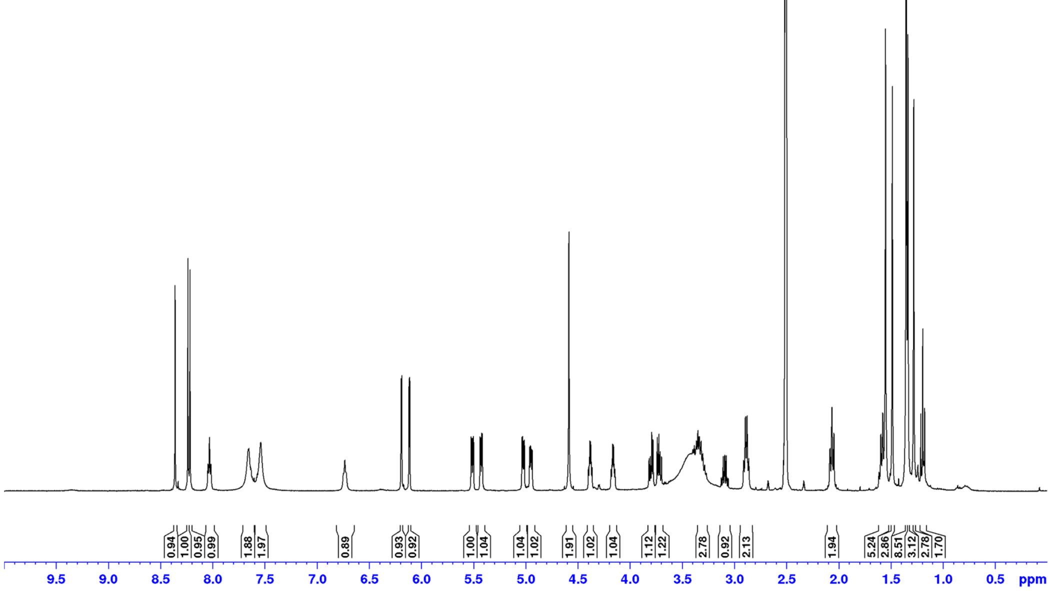

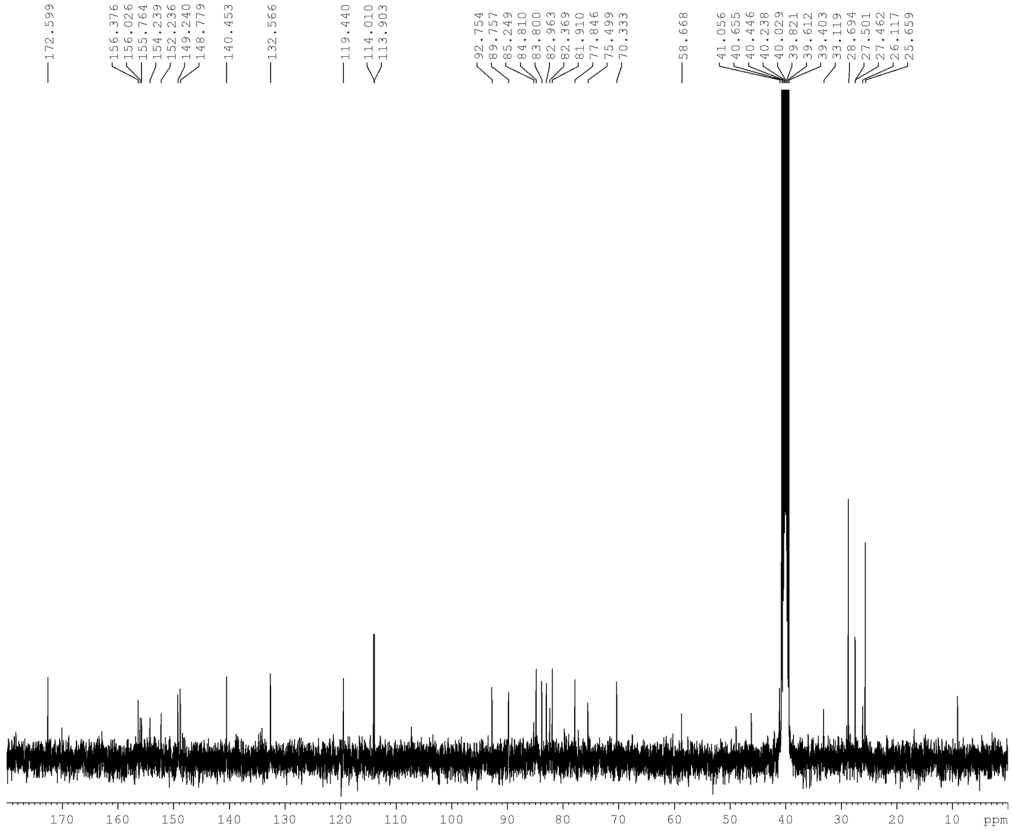

5'-*O*-[3-(5'-(4-Aminobutylamido)-5'-deoxy-adenosin-8-yl)prop-2-yn-1-yl]-adenosine (**4**).

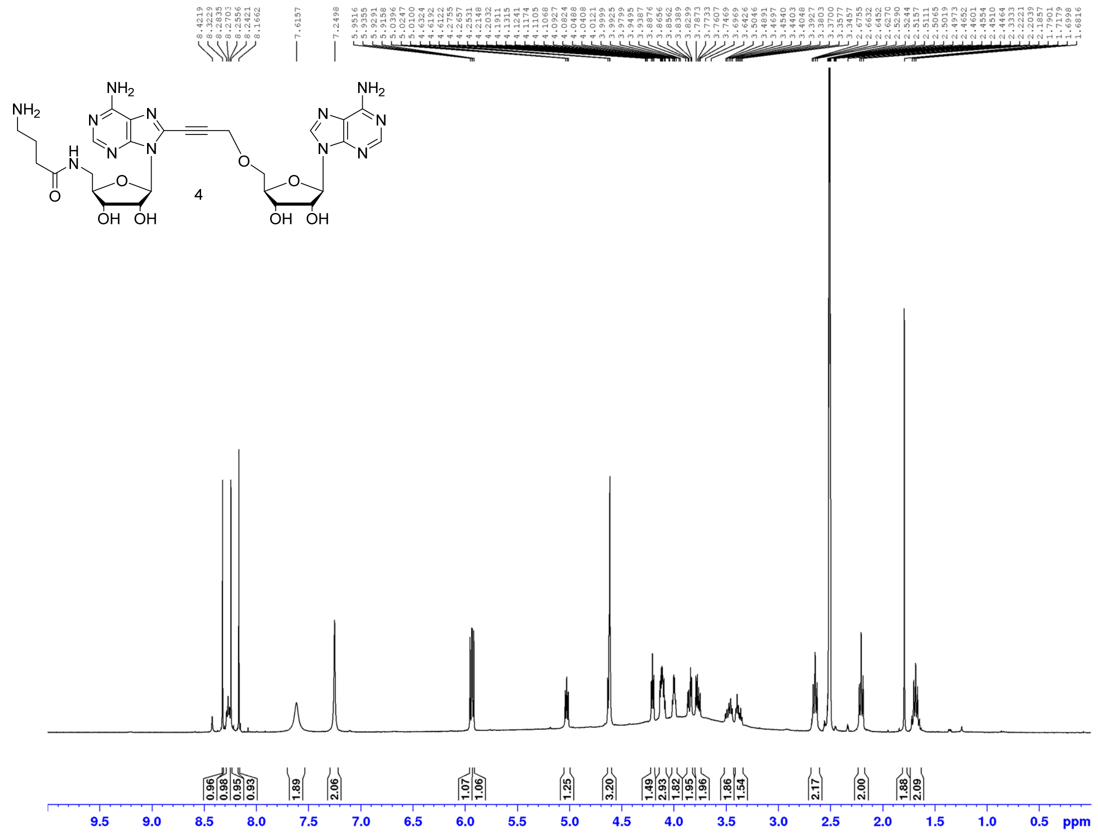

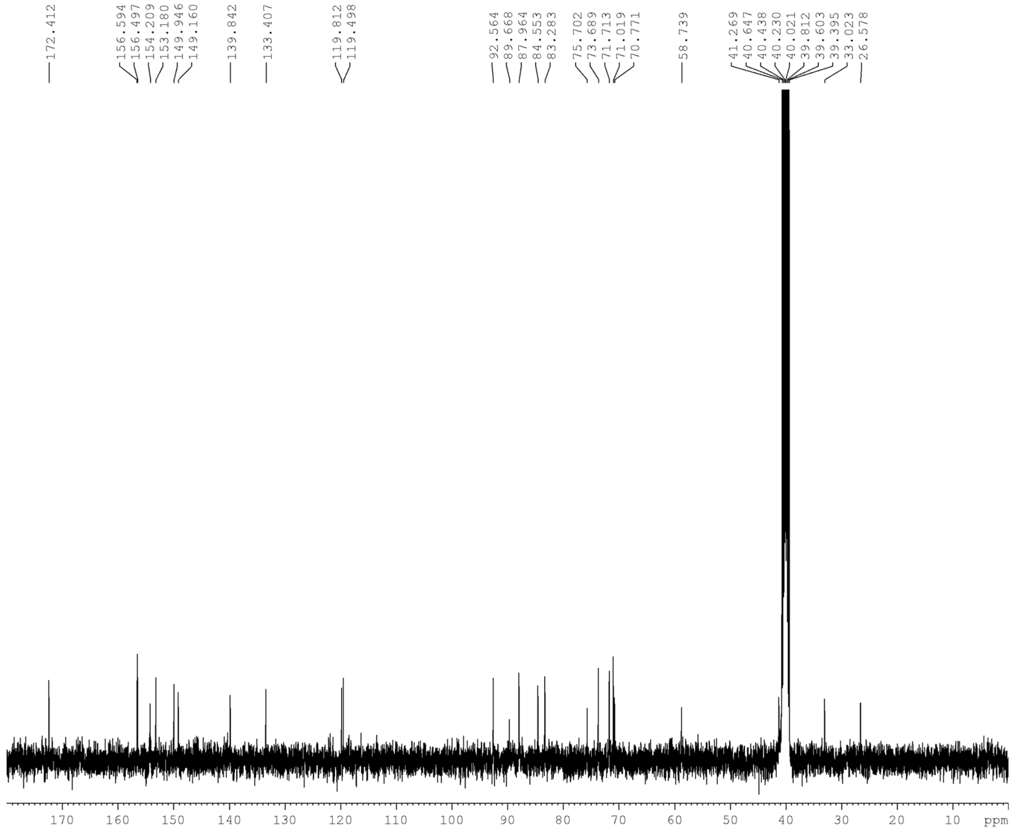

5'-Deoxy-5'-(2-nitrobenzenesulfonamido)-2',3'-*O*-isopropylidene-adenosine (**16**).

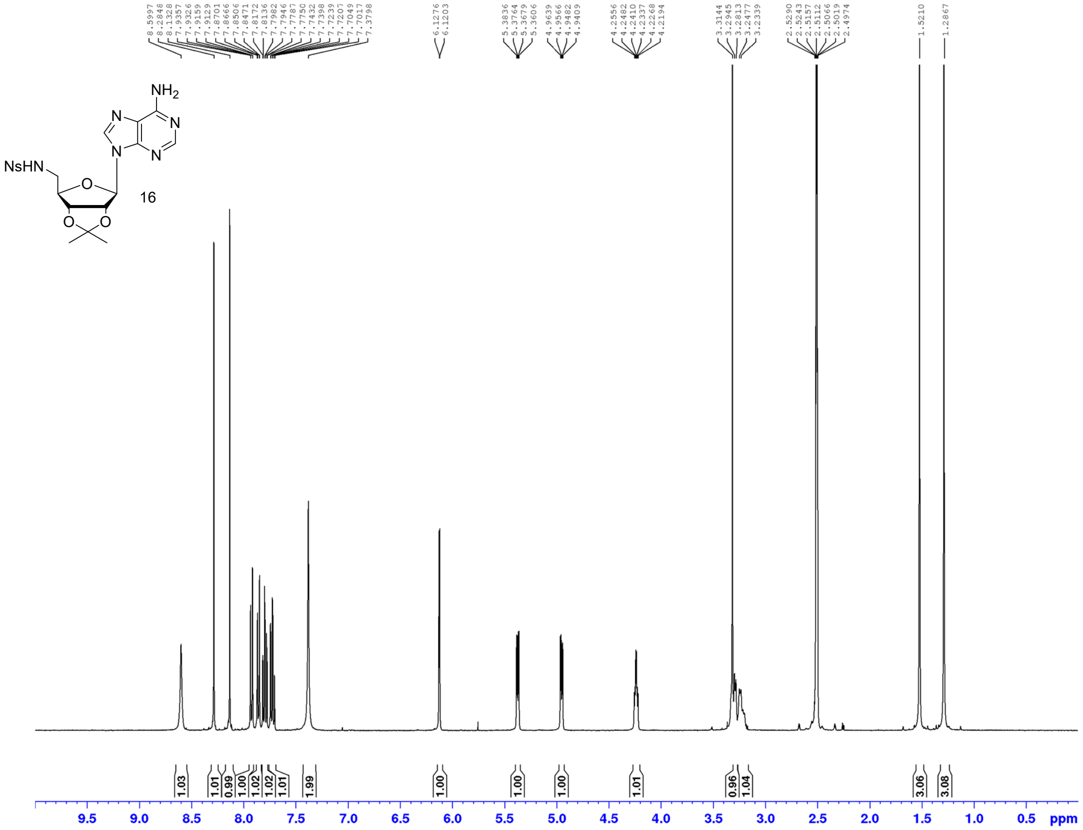

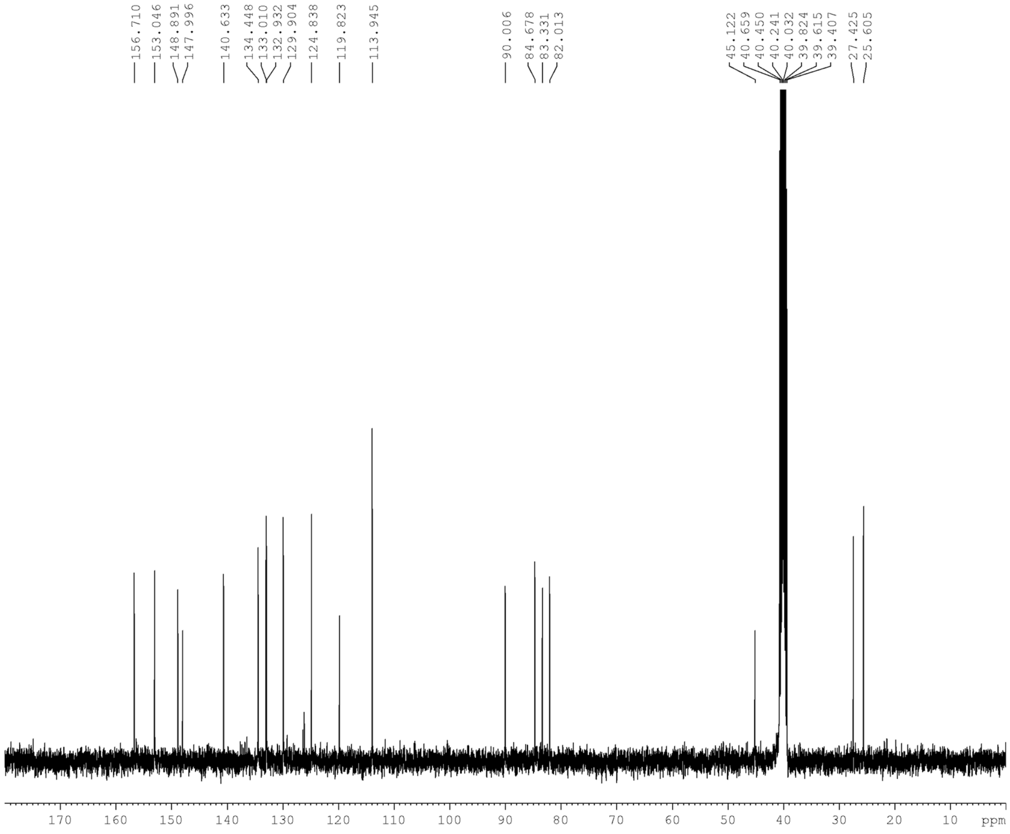

5'-*N*-(*N*-*tert*-Butyloxycarbonyl-aminopropyl)amino-5'-deoxy-2',3'-*O*-isopropylidene-adenosine (**17**).

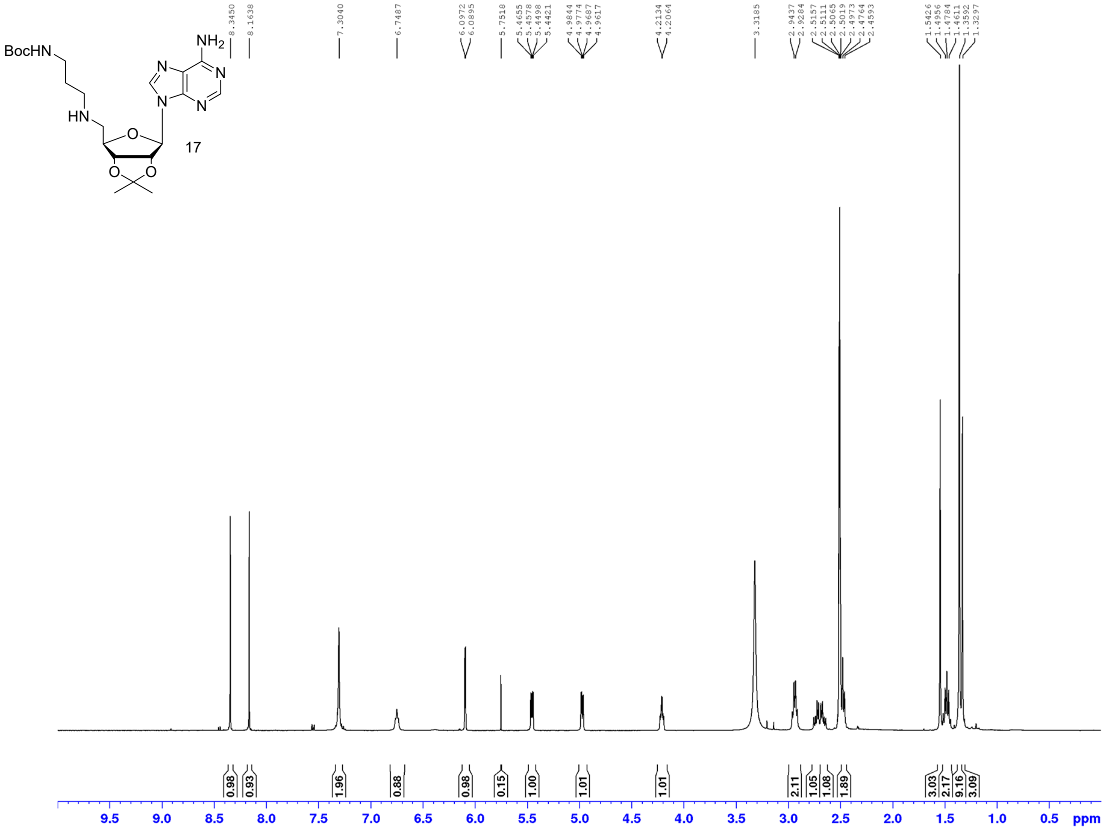

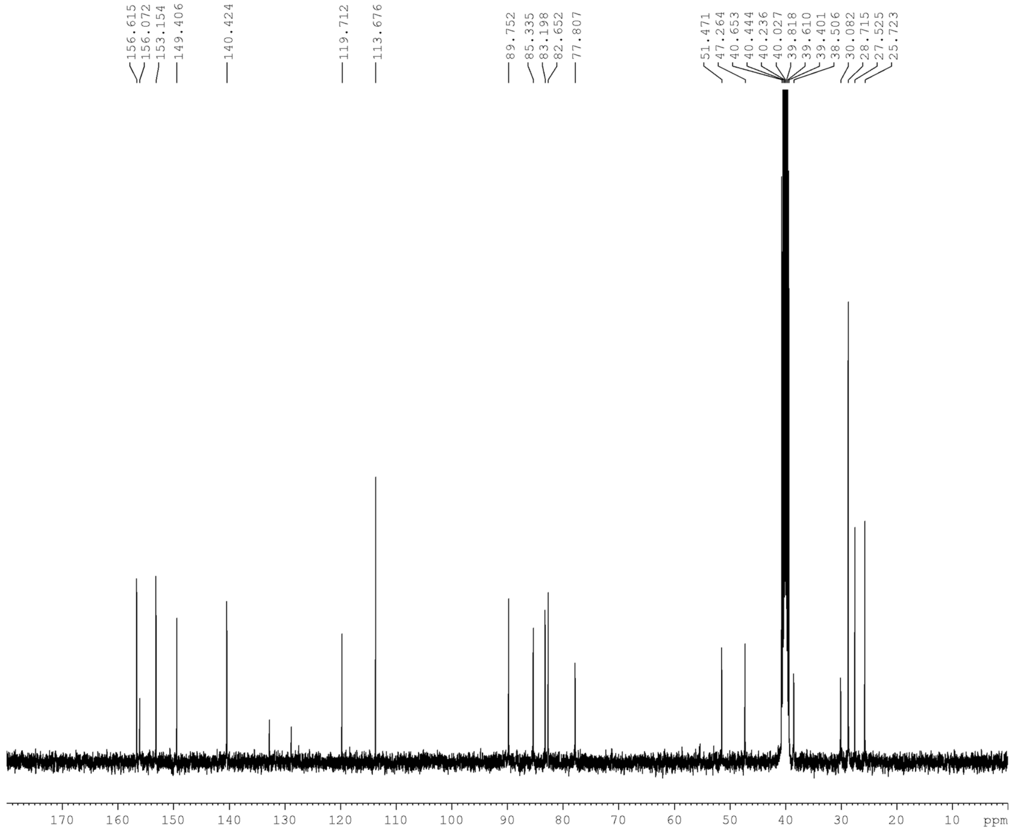

5'-*N*-(*N*-*tert*-Butyloxycarbonyl-aminopropyl)propargylamino-5'-deoxy-2',3'-*O*-isopropylidene-adenosine (**18**).
